# Appropriate exercise level attenuates gut dysbiosis and valeric acid increase to improve neuroplasticity and cognitive function after surgery in mice

**DOI:** 10.1038/s41380-021-01291-y

**Published:** 2021-10-18

**Authors:** Zhongmeng Lai, Weiran Shan, Jun Li, Jia Min, Xianzhang Zeng, Zhiyi Zuo

**Affiliations:** 1 Department of Anesthesiology, University of Virginia, Charlottesville, VA 22908 USA; 2 Department of Anesthesiology, Fujian Medical University Union Hospital, 29 Xin-Quan Road, Fuzhou, Fujian 350001 China; 3 Department of Anesthesiology, First Affiliated Hospital, Nanchang University, Nanchang, Jiangxi 330006 China; 4 Department of Anesthesiology, Second Affiliated Hospital, Harbin Medical University, Harbin, 150001 Heilongjiang China

**Keywords:** Neuroscience, Biochemistry

## Abstract

Postoperative cognitive dysfunction (POCD) affects the outcome of millions of patients each year. Aging is a risk factor for POCD. Here, we showed that surgery induced learning and memory dysfunction in adult mice. Transplantation of feces from surgery mice but not from control mice led to learning and memory impairment in non-surgery mice. Low intensity exercise improved learning and memory in surgery mice. Exercise attenuated surgery-induced neuroinflammation and decrease of gut microbiota diversity. These exercise effects were present in non-exercise mice receiving feces from exercise mice. Exercise reduced valeric acid, a gut microbiota product, in the blood. Valeric acid worsened neuroinflammation, learning and memory in exercise mice with surgery. The downstream effects of exercise included attenuating growth factor decrease, maintaining astrocytes in the A2 phenotypical form possibly via decreasing C3 signaling and improving neuroplasticity. Similar to these results from adult mice, exercise attenuated learning and memory impairment in old mice with surgery. Old mice receiving feces from old exercise mice had better learning and memory than those receiving control old mouse feces. Surgery increased blood valeric acid. Valeric acid blocked exercise effects on learning and memory in old surgery mice. Exercise stabilized gut microbiota, reduced neuroinflammation, attenuated growth factor decrease and preserved neuroplasticity in old mice with surgery. These results provide direct evidence that gut microbiota alteration contributes to POCD development. Valeric acid is a mediator for this effect and a potential target for brain health. Low intensity exercise stabilizes gut microbiota in the presence of insult, such as surgery.

## Highlights

- Exercise reduces post-surgery neuroinflammation and impairment of cognition and neuroplasticity
- Exercise decreases gut microbiota changes and valeric acid increase after surgery
- The effects of exercise on surgery-induced changes are transferable by fecal transplantation
- Valeric acid blocks the beneficial effects of exercise

## Introduction

Postoperative cognitive dysfunction (POCD) affects millions of patients each year in the U.S.A. and is associated with increased mortality and cost of hospitalization [1–4]. Advanced age is an independent risk factor for POCD [1, 2, 5]. Currently the mechanisms for POCD are not fully understood and effective interventions to reduce POCD have not been identified.

Neuroinflammation has been implied in the development of POCD [6, 7]. A surgery on peripheral tissues or organs can induce systemic inflammation, which then is transmitted into the brain to cause neuroinflammation to induce POCD [8, 9]. Gut microbiota has been shown to be involved in regulating inflammation [10]. Interestingly, gut microbiota diversity changes and dysbiosis are associated with learning and memory dysfunction after surgery in animals [11, 12]. Pretreated animals with probiotics improved their cognitive functions [13]. However, direct evidence to suggest the involvement of gut microbiota in POCD has not been reported.

We and others have shown that environmental enrichment reduces learning and memory impairment after surgery [14, 15]. Environment enrichment may enhance the physical activity of animals. A recent study has shown that gut microbiota is important in determining whether human subjects are responders to exercise to improve glucose homeostasis and insulin sensitivity [16]. Exercise has been shown to improve long-term memory of humans [17]. Better pre-surgery exercise ability is associated with less of a decrease in Mini-Mental State Examination scores after cardiac surgery in humans [18]. An animal study has shown that exercise attenuated the enhanced neuroinflammation and impairment of learning and memory after surgery in low-capacity runner rats. Exercise also improved the diversity of gut microbiota of these rats. Although exercise appears to reduce proinflammatory cytokine production in the brain of high capacity rats, it does not attenuate surgery-induced learning and memory impairment in these rats [19]. These results suggest that exercise or exercise capacity is associated with better learning and memory. However, it is not clear whether exercise attenuates POCD, especially in old animals, and whether gut microbiota plays a role in the effects of exercise on POCD.

Based on the above information, we hypothesize that an appropriate level of exercise attenuates neuroinflammation and the impairment of learning and memory after surgery and that these effects are mediated by gut microbiota alterations. To test these hypotheses, we subjected adult and old mice to different levels of exercise. Fecal transplantation was performed to determine the role of gut microbiota in the effects of exercise on POCD development. Left carotid artery exposure was chosen to be the surgical procedure because this procedure is a component of carotid endarterectomy that is commonly performed in elderly patients. In addition, this procedure shall not affect limb functions, which are needed for learning and memory tests, and organ functions for general health. Our results provide direct evidence for the involvement of gut microbiota in POCD. Valeric acid, a product of gut microbiota, plays a critical role in mediating the effects of gut microbiota on learning and memory impairment after surgery.

## Results

### Low intensity exercise prevented surgery-induced cognitive dysfunction, neuroinflammation, and impairment of brain cell generation and dendritic arborization possibly via stabilizing gut microbiota in adult mice

**Fig. 1:**
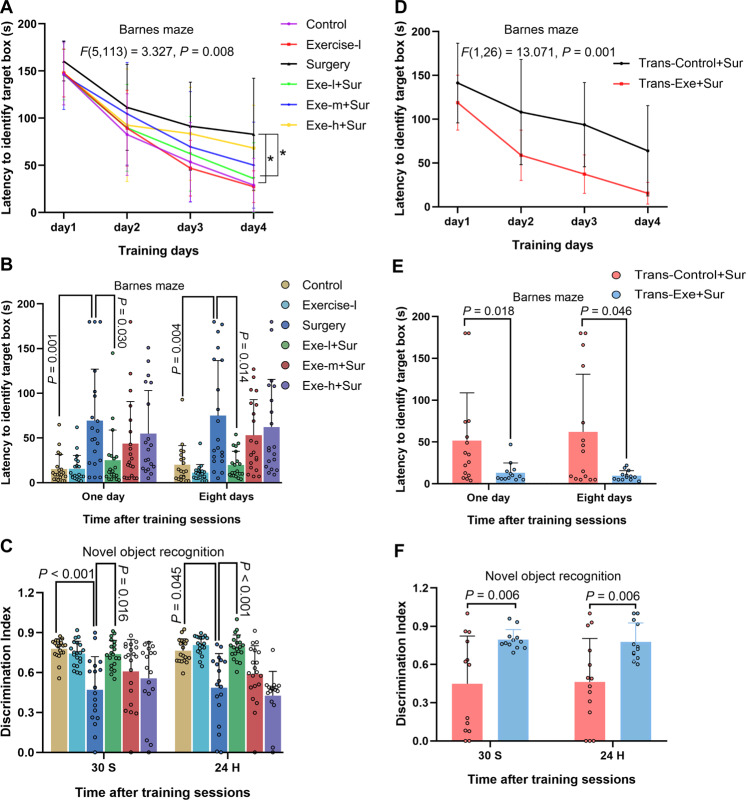
Exercise improved learning and memory. Nine-week old male mice with or without exercise for 4 weeks were subjected to left carotid artery exposure (surgery) under isoflurane anesthesia (panels **A**–**C**). In another experiment, 9-week old mice were treated with antibiotics to eliminate their native gut microbiota and then transplanted with feces from exercise mice (Trans-Exe) or control mice (Trans-Control) 2 weeks before the surgery (panels **D**–**F**). **A**, **D** Training sessions of Barnes maze test. **B**, **E** Memory assessment of Barnes maze test. **C**, **F** Novel object recognition test. Results are mean ± S.D. with (panels **E** and **F**) or without (panels **A** and **D**) presentation of value of individual mouse or median ± interquartile range with presentation of value of individual mouse (panels **B** and **C**) (*n* = 17–20 for panels **A**–**C**, = 13–15 for panels **D**–**F**). Exe-I/Exercise-I low intensity exercise, Exe-m middle intensity exercise, Exe-h high intensity exercise, Sur surgery. *P < 0.05 compared the two curves.

To determine the effect of physical activity on the function and structure of brain, 9-week old mice were subjected to forced mouse treadmill running to 35–40% (low intensity), 55–60% (middle intensity), or 75–80% (high intensity) of their individual maximal exercise capacity, respectively, 5 days a week for 4 weeks before they had left carotid artery exposure for 15 min under isoflurane anesthesia (surgery and anesthesia). Mice were assessed by novel object recognition and Barnes maze tests from one week after the surgery. Mice in all groups took less time to find the target box with more training sessions in the Barnes maze test (Fig. 1A). Surgery was a significant factor to affect the animals to find the target box [F(1, 113) = 7.360, *P* = 0.008] and low intensity exercise reversed this surgery effect in the training sessions of the Barnes maze test [F(1, 76) = 5.278, *P* = 0.024]. Mice with surgery took longer than control mice to find the target box one or eight days after the training sessions. This surgery effect was attenuated by low intensity exercise but not by middle or high intensity exercise (Fig. 1B). Mice with surgery spent less time with novel object than control mice in the novel object recognition test and this decreased time was reversed by low intensity exercise but not by middle or high intensity exercise no matter when the test was performed either 30 s or 24 h after the initial exploration with two objects (familiarization phase) (Fig. 1C). Various lengths of delay between the familiarization and test phases (from 10 s to 24 h) have been used previously. The memorization after short and long delays may involve perirhinal cortex and hippocampus, respectively [20]. Our results suggest that surgery induces learning and memory dysfunction and that low intensity exercise but not middle and high intensity exercise prevents these surgery effects.

As an initial step to determine whether gut microbiota played a role in the effects of exercise on learning and memory, mice transplanted with feces from control mice or from mice with low intensity exercise were subjected to surgery. Transplantation with feces from exercise mice reduced the amount of time to identify the target box in the training sessions [F(1, 26) = 13.071, *P* = 0.001] and one or eight days after training sessions (Fig. 1D, E). Mice transplanted with feces from exercise mice spent more time with novel object than mice transplanted with feces from control mice (Fig. 1F). These results suggest that gut microbiota mediates the beneficial effects of low intensity exercise. This level of exercise was used for mice receiving exercise conditioning in the following experiments and was referred as exercise for simplicity.

**Fig. 2:**
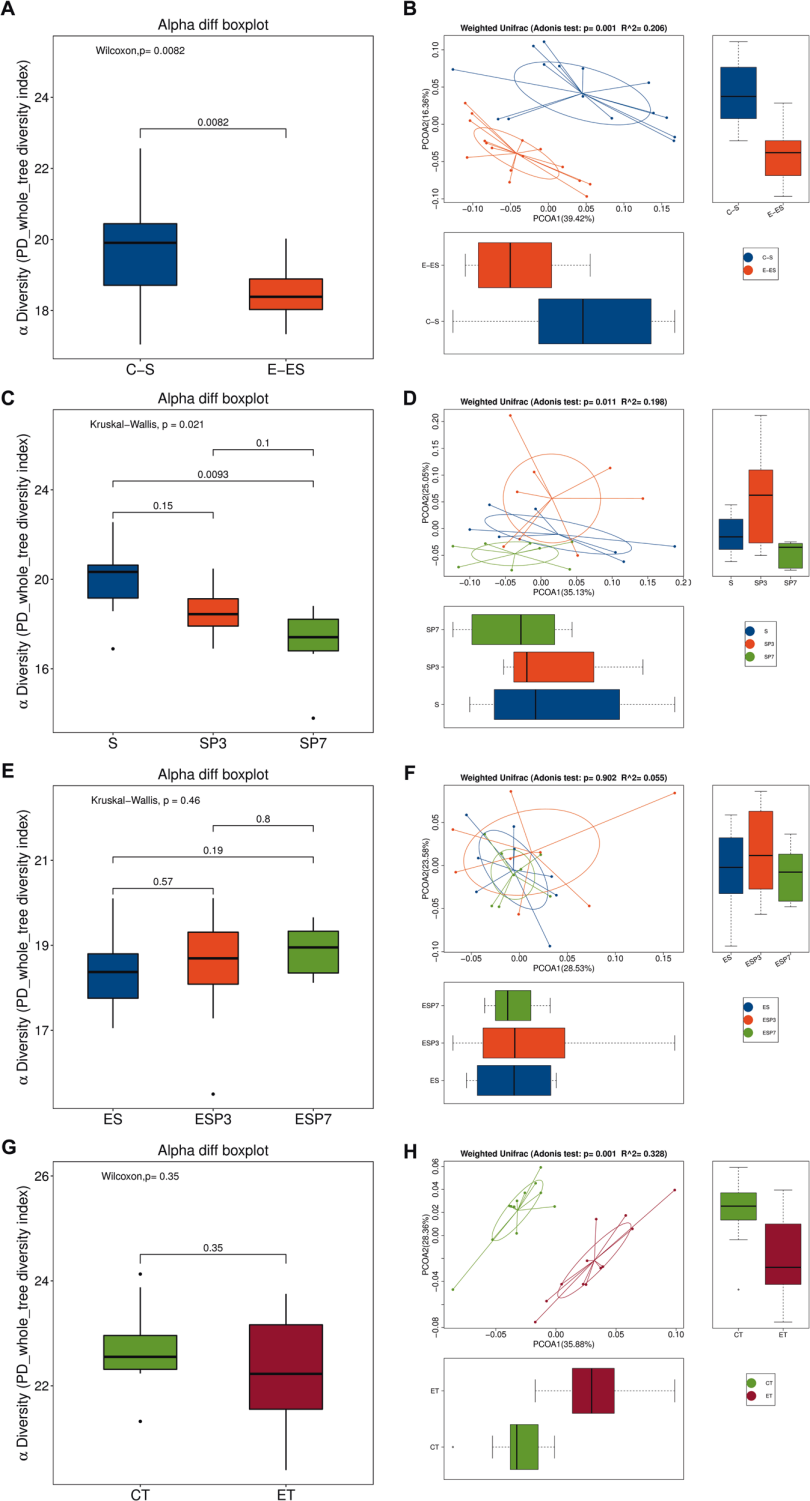
Exercise stabilized gut microbiota of mice with surgery. Nine-week old male mice with or without exercise for 4 weeks were subjected to left carotid artery exposure (surgery) under isoflurane anesthesia. In another experiment, 9-week old mice were treated with antibiotics to eliminate their native gut microbiota and then transplanted with feces from exercise mice (ET) or control mice (CT). Presentation of α diversity is in (panels **A**, **C**, **E** and **G**). Presentation of β diversity is in (panels **B**, **D**, **F** and **H**). *n* = 16 for (panels **A** and **B**, and = 8 for other panels). C-S: control mouse samples harvested before surgery, E-ES: exercise mouse samples harvested before surgery, S: surgery mouse samples harvested just before surgery, SP3: surgery mouse samples harvested on post-surgery day 3, SP7: surgery mouse samples harvested on post-surgery day 7, ES: exercise mouse samples harvested just before surgery, ESP3: exercise mouse samples harvested on post-surgery day 3, ESP7: exercise mouse samples harvested on post-surgery day 7, CT: samples harvested from mice transplanted with feces from control mice, ET: samples harvested from mice transplanted with feces from exercise mice.

Exercise modified the diversity of gut microbiota within one sample (α diversity) and the difference in diversity among samples (β diversity) (Fig. 2A, B, and Supplementary Fig. 1a–c). Exercise increased the abundance of some bacteria, such as *Bacteroidales and Alistipes*. Control mice had abundant *lactobacilliaceae and lactobacillus* because the linear discriminant analysis (LDA) scores for the comparisons of these bacteria between control and exercise mice were all >3.5 (the direction can be negative or positive) (Supplementary Fig. S1d), a threshold indicating difference in the abundance of bacteria in samples from different experimental conditions [21]. LDA was performed after the difference among samples from different experimental conditions was determined to be significant by Kruskal–Wallis test by rank per LDA effect size analysis protocol. Mice had a reduced diversity of gut microbiota and the difference in diversity among samples 7 days after surgery. This reduction was attenuated in exercise mice (Fig. 2C–F and Supplementary Fig. S2). Mice receiving fecal transplantation had treatment of the mixture of antibiotics to eliminate their native gut microbiota prior to the transplantation. This treatment nearly abolished the gut microbiota in these mice because the bacterial DNA in the feces was barely detectable (Supplementary Fig. S3). Mice transplanted with feces from exercise mice also had a reduced difference in diversity among samples (Fig. 2G, H and Supplementary Fig. S4a–c). Gut microbiota of mice transplanted with feces from exercise mice contained an abundant amount of *Alistipes*. Mice transplanted with feces of control mice contained a large amount of *lactobacilliaceae and lactobacillus* (Supplementary Fig. S4d). These results suggest that exercise changed gut microbiota with increased abundance in phylum Bacteroidetes, such as *Bacteroidales and Alistipes*, and decreased abundance in phylum Firmicutes, such as *lactobacilliaceae and lactobacillus*. Exercise also stabilizes gut microbiota after surgery.

Consistent with the pattern of microbiota in mice with surgery, mice transplanted with feces of surgery mice had reduced diversity of gut microbiota compared with mice transplanted with feces of control mice. Mice receiving surgery mouse feces had decreased abundance in *Bacteroidales and Alistipes* compared with mice receiving control mouse feces (Supplementary Fig. S5). These results suggest that altered microbiota in surgery mice is successfully transferred to non-surgery mice. Mice receiving surgery mouse feces took longer than mice receiving control mouse feces to identify the target box in Barnes maze one day after the training sessions. The mice receiving surgery mouse feces also spent less time with novel object than mice receiving control mouse feces in the novel object recognition test. However, there was no difference among control mice (naïve mice), mice receiving antibiotics and mice receiving control mouse feces in the performance of Barnes maze and novel object recognition tests (Supplementary Fig. S6). These results indicate a role of microbiota alteration in learning and memory dysfunction after surgery.

**Fig. 3:**
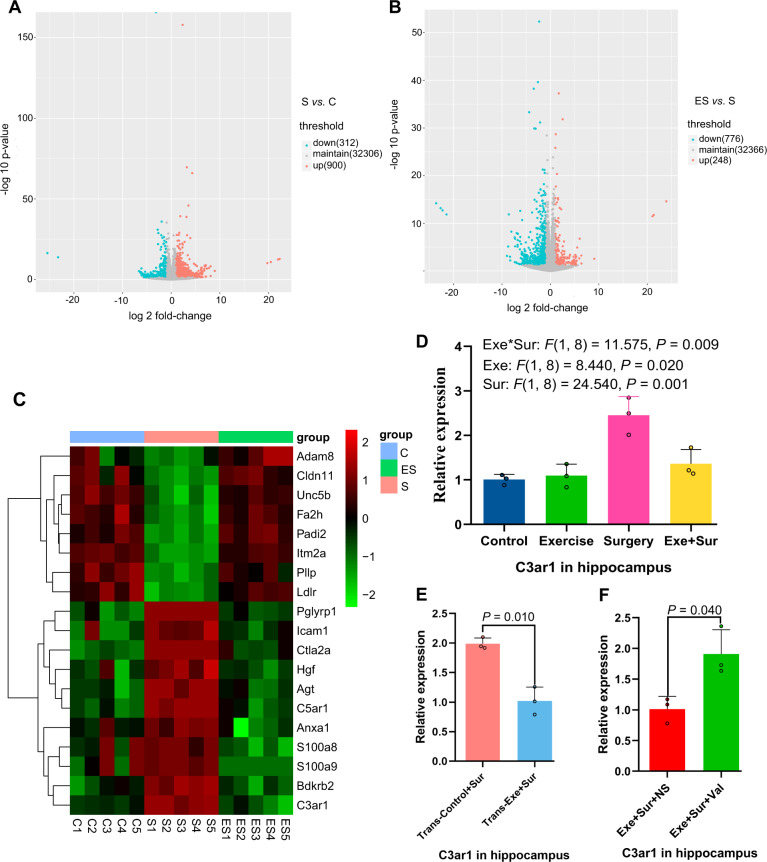
mRNA expression profile of mice. Hippocampal samples were subjected to RNA-seq analysis. **A**, **B** Volcano plot. **C** Heatmap of mRNA abundance of genes whose expression was different among the three groups of animals. **D**–**F** Quantitative data of real-time PCR analysis. Results in (panels **D**–**F**) are mean ± S.D. with presentation of value of individual mouse (*n* = 5 for panels **A**–**C**, and = 3 for panels **D**–**F**). C control, S or Sur surgery, ES or Exe+Sur exercise plus surgery, Trans-Control: mice transplanted with feces from control mice, Trans-Exe: mice transplanted with feces from exercise mice, NS normal saline, Val valeric acid.

**Fig. 4:**
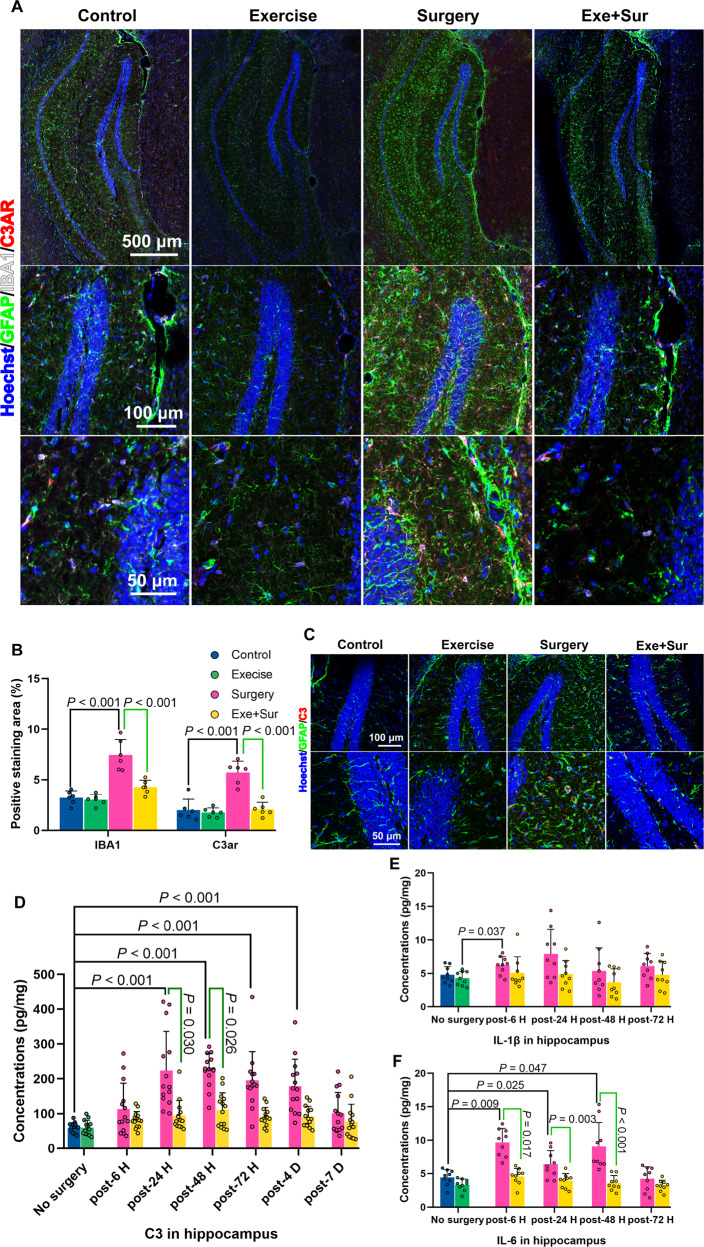
Exercise attenuated surgery-induced immune and inflammatory responses. Hippocampus was harvested at various times after surgery for immunostaining or ELISA. **A** Representative Iba-1 and C3ar immunostaining images of hippocampus harvested 48 h after surgery. **B** Quantification of Iba-1 and C3ar immunostaining of hippocampus harvested 48 h after surgery. **C** Representative C3 immunostaining images of hippocampus harvested 48 h after surgery. **D** Quantification of C3 by ELISA. **E** IL-1β quantified by ELISA. **F** IL-6 quantified by ELISA. Results in (panels **B**, **E** and **F**) are mean ± S.D. with presentation of value of individual mouse and result in (panels **D**) is median ± interquartile range with presentation of value of individual mouse (*n* = 6 for panel **B**,  = 14 for panel **D**,  = 9 for panels **E** and **F**). Exe exercise, Sur surgery, Post post-surgery.

As an initial step to identify possible molecules downstream of low intensity exercise and gut microbiota for regulating learning and memory, we performed RNA-seq analysis. Surgery and exercise altered the profiles of mRNA expression in the hippocampus (Fig. 3A, B), a brain region involved in learning and memory [6, 22]. The genes with most changes by surgery and exercise were displayed in Fig. 3C. Among them, the mRNA of complement 3a receptor 1 (C3ar1) was increased by surgery and this increase was attenuated by exercise based on a two-way analysis of variance with surgery and exercise as two independent factors (Fig. 3C, D). The mRNA expression of C3ar1 after surgery was decreased in mice transplanted with feces from exercise mice (Fig. 3E). Consistent with this mRNA result, surgery increased C3ar protein (Fig. 4A, B). C3, a ligand for C3ar [23], was also increased by surgery and this increase was reduced by exercise (Fig. 4C, D). Consistent with the ideas that C3 signaling is important in immunomodulation [24] and that surgery can induce neuroinflammation [8], surgery increased ionized calcium binding adaptor molecule 1 (Iba-1) and interleukin (IL)−6 expression in the hippocampus and exercise attenuated this increase (Fig. 4A, B, F). However, surgery and exercise did not significantly change the expression of IL-1β (Fig. 4E). These results suggest that surgery induces immune and inflammatory responses and that exercise blocks these responses.

**Fig. 5:**
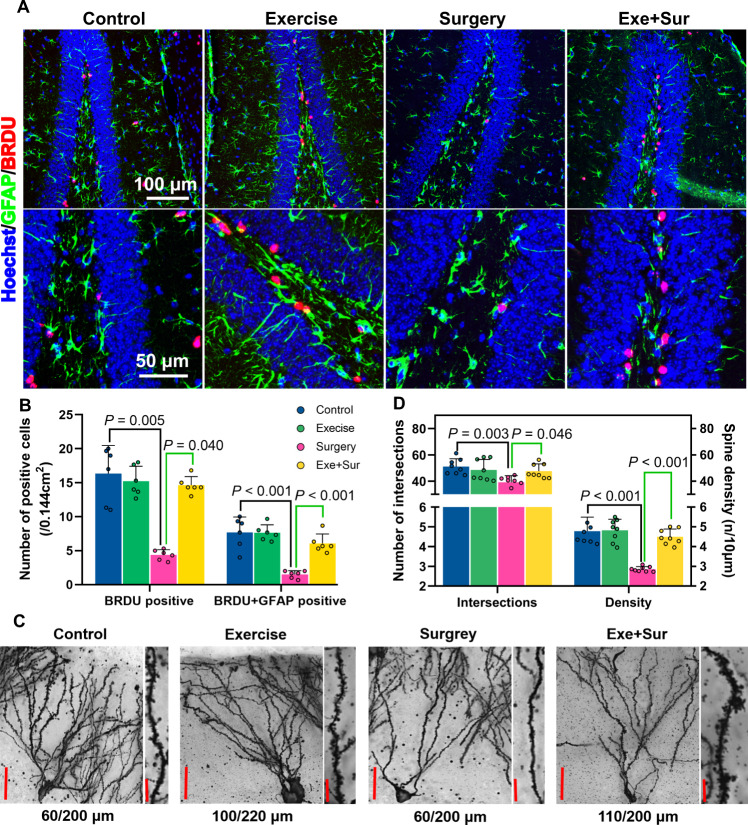
Exercise attenuated surgery-induced decrease of brain cell genesis and dendritic arborization in young adult mice. Brain was harvested 19 days after surgery for immunostaining or Golgi staining. **A** Representative GFAP and BrdU immunostaining images of hippocampus. **B** Quantification of GFAP and BrdU positively stained cells in the hippocampus. **C** Representative Golgi staining images of hippocampus. **D** Quantification of intersections among the dendritic branches and spine density in the hippocampus. Results in (panels **B** and **D**) are mean ± S.D. with presentation of value of individual mouse (*n* = 6 for panel **B**,  = 8 for panel **D**). Exe exercise, Sur surgery.

Learning and memory often require brain structural modification, such as brain cell genesis and dendritic arborization [14, 22]. Surgery reduced brain cell genesis assessed by 5′-bromo-2′- deoxyuridine (BrdU) incorporation. The decreased newly-generated cells included glial fibrillary acidic protein (GFAP)-positive cells. Exercise attenuated this surgery effect (Fig. 5A, B). Surgery also reduced intersections among dendritic branches and spine densities. These surgical effects were attenuated by exercise (Fig. 5C, D). These results suggest that surgery impairs dendritic arborization and that exercise blocks this impairment.

### Critical role of valeric acid, C3 signaling and glial cell-derived neurotrophic factor (GDNF) in mediating the effects of low intensity exercise on POCD via gut microbiota in adult mice

**Fig. 6:**
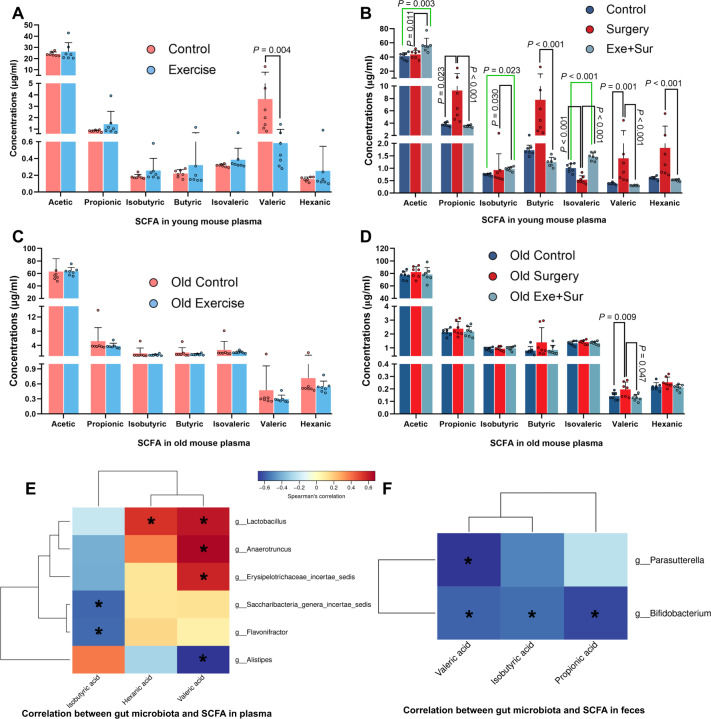
Exercise attenuated surgery-induced changes in short chain fatty acids (SCFAs). Blood was harvested either 4 weeks after the onset of exercise protocol (for panels **A**, **C**, **E** and **F**) or 7 days after surgery (panels **B** and **D**) from 13 and 14-week old (young mice) or 19-month old mice (old mice). **A** Blood SCFAs just before the surgery in young mice. **B** Blood SCFAs 7 days after the surgery in young mice. **C** Blood SCFAs just before the surgery in old mice. **D** Blood SCFAs 7 days after the surgery in old mice. **E** Correlation presentation between gut bacteria and blood SCFA concentrations in young adult mice. **F** Correlation presentation between gut bacteria and fecal SCFA concentrations in young adult mice. Results in (panels **A**–**D**) are median ± interquartile range (for propionic acid, butyric acid, valeric acid and hexanic acid) with presentation of value of individual mouse or mean ± S.D. (for other SCFAs) with presentation of value of individual mouse (*n* = 7 for all panels). Exe exercise, Sur surgery. In (panels **E** and **F**), *P < 0.05 for the correlation, red: positive correlation, blue: negative correlation.

To identify possible mediators for the effects of gut microbiota on learning and memory after surgery and to determine whether gut microbiota mediates the effects of exercise on surgery-induced neuroinflammatory responses and impaired dendritic arborization, short chain fatty acids (SCFAs) in the blood were measured. SCFAs are products of gut microbiota. Valeric acid was the only SCFA whose concentrations in the blood were decreased in mice with exercise prior to surgery (Fig. 6A). Valeric acid was increased by surgery and this increase was attenuated by exercise in mice with surgery (Fig. 6B). In addition to valeric acid, exercise decreased propionic acid, butyric acid and hexanic acid and increased acetic acid, isobutyric acid and isovaleric acid in surgery mice. The increase of valeric acid in the blood was positively associated with *lactobacillus* and *anaerotruncus* and was negatively associated with *Alistipes* when the results of control mice and exercise mice were analyzed together (Fig. 6E). Consistent with this correlation and as described above, exercise reduced *lactobacillus* and increased *Alistipes* (Supplementary Fig. S1d). Interestingly, the bacteria that were associated with decreased valeric acid in the feces (Fig. 6F) did not include the bacterium that was associated with decreased valeric acid in the blood, suggesting additional mechanisms for regulating the absorption of valeric acid in the gut into the blood or the metabolism of valeric acid in the feces during the transporting of feces to rectum.

Mice transplanted with feces from exercise mice had lower blood valeric acid than mice transplanted with feces from control mice (Supplementary Fig. S7a). In another set of experiments, we reproduced the findings that exercise reduced valeric acid, butyric acid and hexanic acid in mice with surgery. Intraperitoneal injection of valeric acid attenuated this reduction caused by exercise (Supplementary Fig. S7b). Interestingly, exercise did not change the concentrations of SCFAs in the feces (Supplementary Fig. S7c). SCFAs in the feces of mice transplanted with feces from exercise mice were not different from those of mice transplanted with feces from control mice (Supplementary Fig. S7d). These results support the idea that measuring fecal SCFAs may not be useful to reflect the concentrations of SCFAs in the blood.

**Fig. 7:**
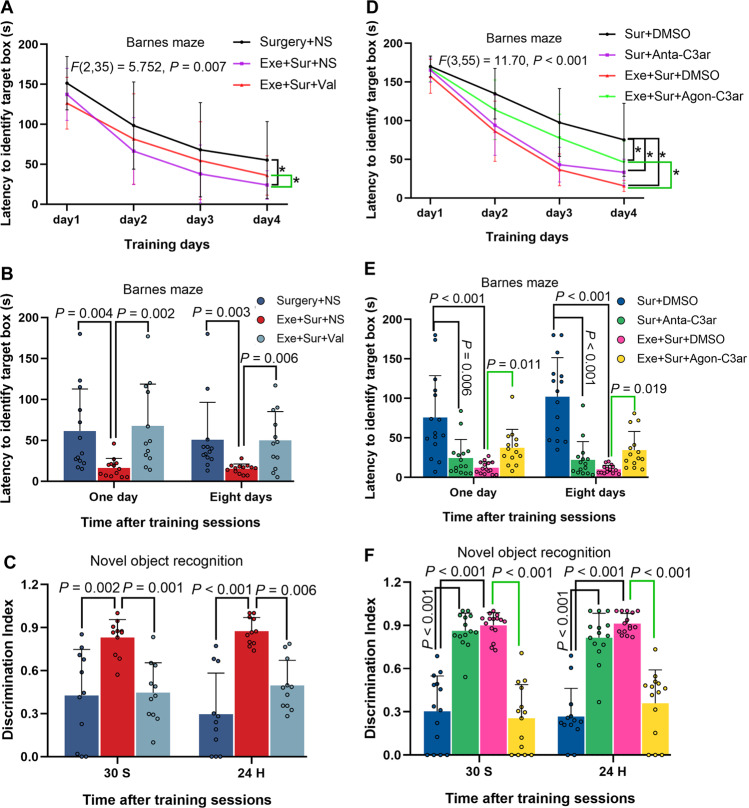
Role of valeric acid and C3 signaling in exercise attenuation on surgery-induced learning and memory impairment. Nine-week old male mice with or without exercise for 4 weeks in the presence or absence of intraperitoneal injection of valeric acid (one injection per week) were subjected to left carotid artery exposure (surgery) under isoflurane anesthesia (panels **A**–**C**). In another experiment, 9-week old mice with or without exercise for 4 weeks were subjected to surgery and received intracerebroventricular injection of a C3 agonist or SB290157, a C3ar antagonist, at 0, 24, 48 and 72 h after surgery (panels **D**–**F**). **A**, **D** Training sessions of Barnes maze test. **B**, **E** Memory assessment of Barnes maze test. **C**, **F** Novel object recognition test. Results are mean ± S.D. in (panels **A** and **D**) and median ± interquartile range with presentation of value of individual mouse in panels **B**, **C**, **E** and **F** (*n* = 11–13 for panels **A**–**C**,  = 13–15 for panels **D**–**F**). Exe exercise, Sur surgery, NS normal saline, DMSO dimethylsulfoxide, Val valeric acid, Anta-C3ar C3ar antagonist, Agon-C3ar C3ar agonist. *P < 0.05 compared the two curves.

We decided to study the potential role of valeric acid in participating in exercise effects because its concentration was decreased by exercise under baseline condition or after surgery. Consistent with the findings described above, mice with surgery took longer than control mice to identify target box in the Barnes maze test and this effect was attenuated by exercise. Intraperitoneal injection of valeric acid reversed the effects of exercise (Fig. 7A, B). Similarly, exercise blocked the impairment of surgical mice in novel object recognition test and this protection of exercise was abolished by intraperitoneal injection of valeric acid (Fig. 7C). However, intracerebroventricular injection of valeric acid at 1/50 dose of intraperitoneal injection did not affect the performance of mice in the Barnes maze and novel object recognition tests (Supplementary Fig. S8). Taking together, these results suggest that reducing blood valeric acid may be a mechanism for exercise-induced protection against learning and memory impairment after surgery.

Consistent with the results of learning and memory, mice transplanted with feces from exercise mice had reduced C3ar1, C3, Iba-1, IL-1β and IL-6 in the hippocampus compared with mice transplanted with feces from control mice after surgery (Supplementary Fig. S9a, b, d, f, h). Exercise reduced C3, C3ar, Iba-1 and IL-6 in the hippocampus and this reduction was blocked by intraperitoneal injection of valeric acid in mice with surgery (Fig. 3G and Supplementary Fig. S9a, c, e, g, i). These results suggest that reducing blood valeric acid may be a mechanism for exercise-induced protection against immune and neuroinflammatory responses in the brain after surgery.

To determine whether C3 signaling played a role in the learning and memory impairment after surgery, mice received intracerebroventricular injection of a C3ar antagonist or agonist. Surgery mice received SB290157, a C3ar antagonist [25], took less time than surgery mice received solvent injection to identify target box in Barnes maze. C3ar agonist increased the time for surgery mice with exercise to identify the target box (Fig. 7D, E). SB290157 also improved the performance of surgery mice in novel object recognition test. C3ar agonist worsened the performance of exercise mice with surgery in novel object recognition test (Fig. 7F). These results suggest the role of C3 signaling in the development of POCD.

Mice transplanted with feces from exercise mice after surgery had more newly generated brain cells, intersections among dendritic branches and spine density than mice transplanted with feces from control mice (Supplementary Fig. S10a, b, d, f). Exercise attenuated the decrease of newly generated brain cells, intersections among dendritic branches and spine density in the hippocampus and this attenuation was blocked by intraperitoneal injection of valeric acid in mice with surgery (Supplementary Fig. S10a, c, e, g). In addition, surgery reduced the expression of postsynaptic density protein 95 (PSD95) and synapsin-1, two synaptic proteins. This decrease was attenuated by exercise. Mice transplanted with feces from exercise mice after surgery had increased PSD95 and synapsin-1 compared with mice transplanted with feces from control mice (Supplementary Fig. S11). Together, these results suggest that reducing blood valeric acid via altering gut microbiota may be a mechanism for exercise-induced protection against the reduction of brain cell genesis and dendritic arborization after surgery.

**Fig. 8:**
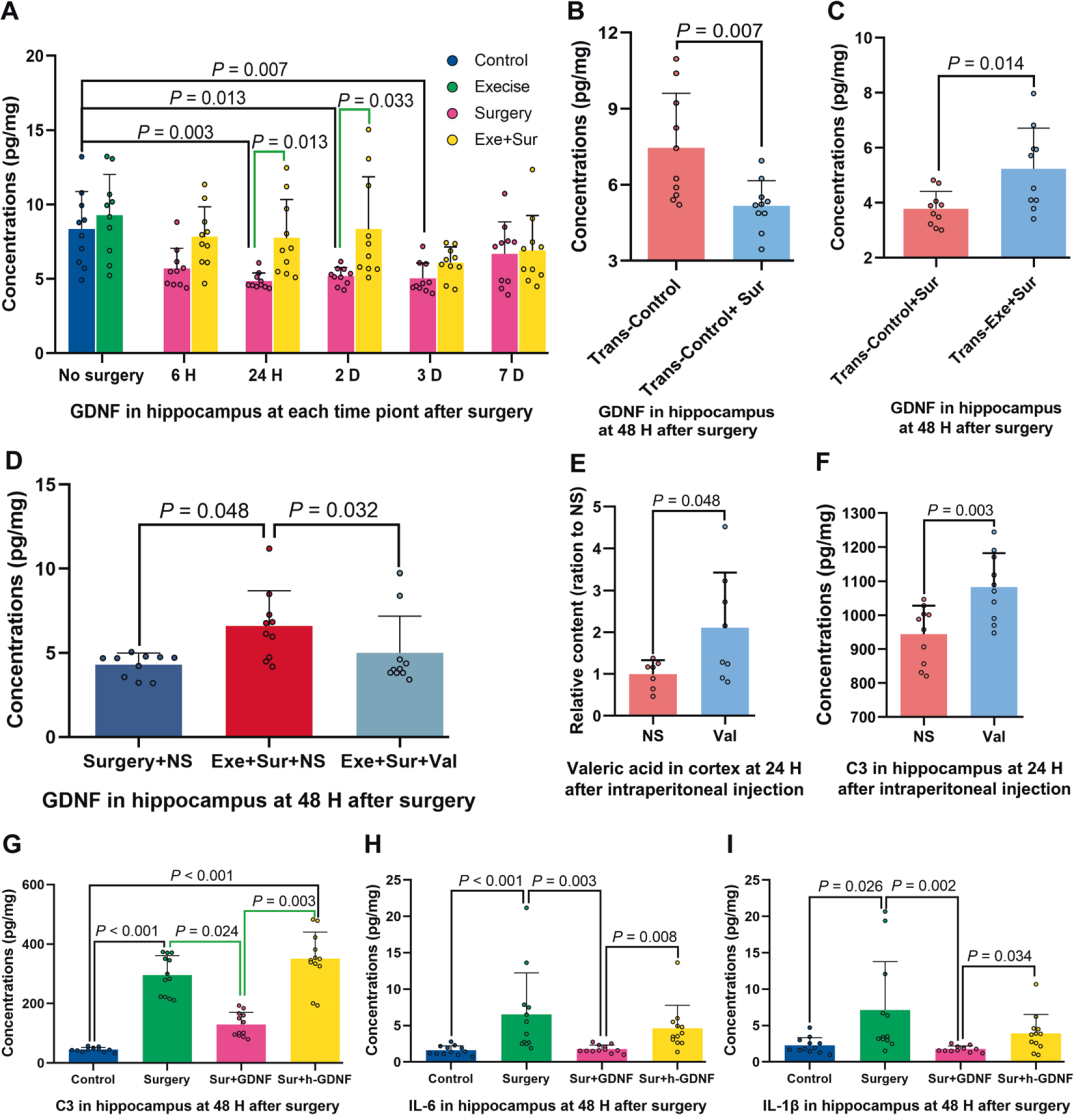
Exercise via regulating gut microbiota attenuated surgery-induced GDNF decrease and GDNF participated in regulation of immune and inflammatory responses after surgery. Nine-week old male mice with or without exercise for 4 weeks in the presence or absence of intraperitoneal injection of valeric acid (one injection per week) were subjected to left carotid artery exposure (surgery) under isoflurane anesthesia (panels **A** and **D**). In second experiment, 9-week old mice were treated with antibiotics to eliminate their native gut microbiota and then transplanted with feces from control mice (Trans-Control) before the surgery (panel **B**). In third experiment, 9-week old mice were treated with antibiotics to eliminate their native gut microbiota and then transplanted with feces of exercise mice (Trans-Exe) or control mice (Trans-Control) before the surgery (panel **C**). In fourth experiment, 9-week old mice received intraperitoneal injection of valeric acid or normal saline (panels **E** and **F**). In fifth experiments, 9-week old mice were subjected to surgery and received intracerebroventricular injection of GDNF immediately after surgery (panels **G**–**I**). **A**–**D** GDNF concentrations in the hippocampus. **E** Valeric acid in the cerebral cortex. **F**, **G** C3 concentrations in the hippocampus. **H** IL−6 concentrations in the hippocampus. **i** IL-1β concentrations in the hippocampus. Results in (panels **A**, **B**, **D** and **G**) are median ± interquartile range with presentation of value of individual mouse and results in other panels are mean ± S.D. with presentation of value of individual mouse (*n* = 10 for panels **A**–**D** and panel **F**, = 7–8 for panel **E**, = 12 for panels **G**–**I**). Exe exercise, Sur surgery, NS normal saline, Val valeric acid.

Our previous studies have shown that the reduction of GDNF plays an important role in POCD [6, 26]. Consistent with our previous finding [6, 26], surgery decreased GDNF in the hippocampus. Exercise blocked this decrease (Fig. 8A). Surgery reduced GDNF in the hippocampus of mice transplanted with feces from control mice (Fig. 8B). Mice transplanted with feces from exercise mice had higher GDNF concentrations in the hippocampus than mice transplanted with feces from control mice after surgery (Fig. 8C). Intraperitoneal injection of valeric acid decreased GDNF levels in the hippocampus of exercise mice with surgery (Fig. 8D). This injection increased the amount of valeric acid and C3 in the brain (Fig. 8E, F). These results, along with our previous findings that GDNF plays a role in the development of POCD [6, 26], suggest that GDNF may be a molecule downstream of the alteration of gut microbiota for the effects of exercise to regulate the development of POCD. To support this idea, GDNF but not the heat-inactivated GDNF reduced C3, IL-6 and IL-1β levels in the hippocampus after surgery (Fig. 8G–I), suggesting that GDNF attenuates surgery-induced immune and inflammatory responses, which are considered to be important for POCD development [6, 7].

### Gut microbiota and valeric acid played an important role in exercise-induced attenuation of neuroinflammation and impairment of learning, memory, brain cell generation and dendritic arborization after surgery in old mice

**Fig. 9:**
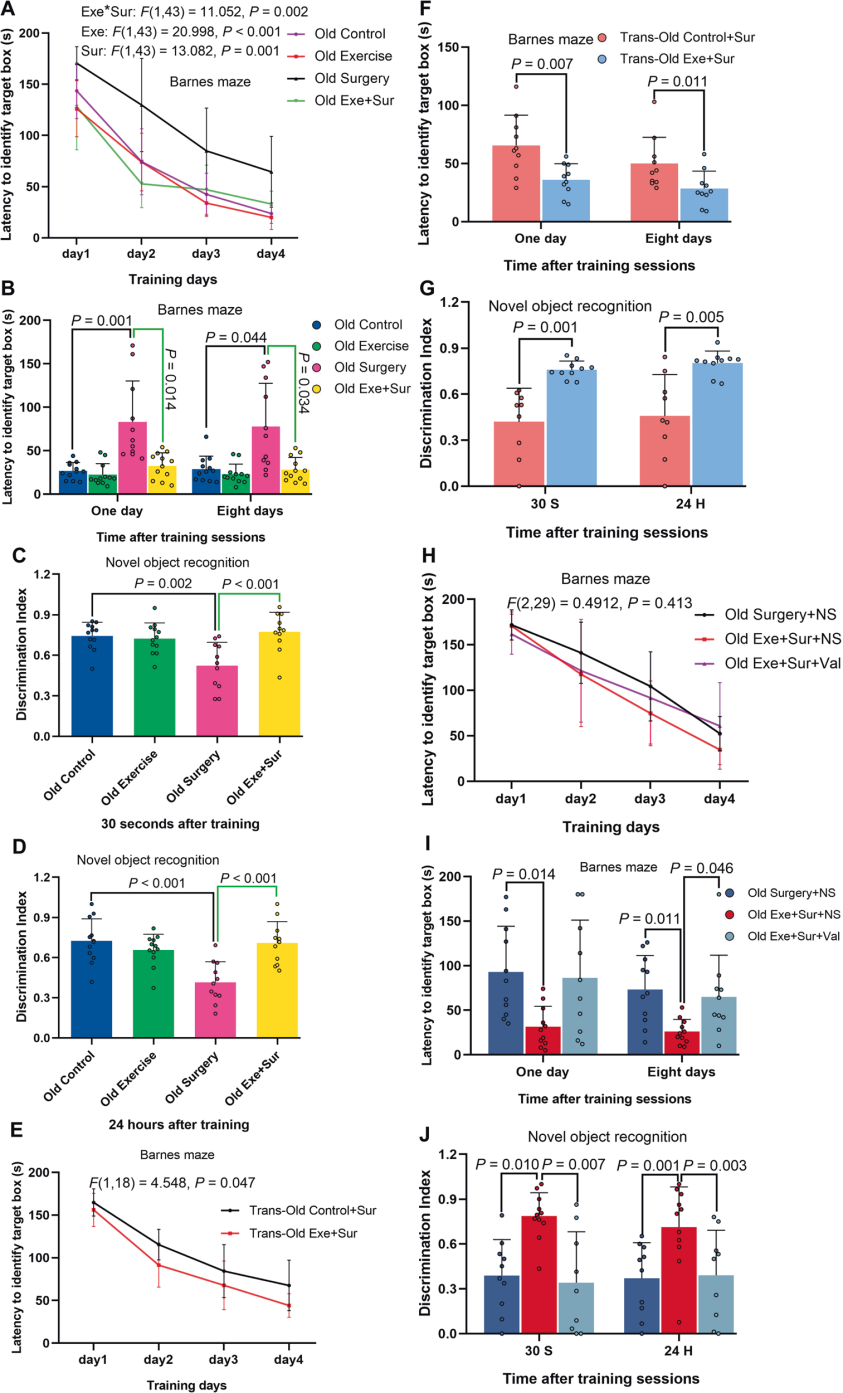
Exercise via regulating gut microbiota and blood valeric acid attenuated surgery-induced learning and memory impairment in old mice. Nineteen-month old male mice with or without exercise for 4 weeks were subjected to left carotid artery exposure (surgery) under isoflurane anesthesia (panels **A**–**D**). In second experiment, 19-month old mice were treated with antibiotics to eliminate their native gut microbiota and then transplanted with feces from exercise mice (Trans-Exe) or control mice (Trans-Control) before the surgery (panels **E**–**G**). In third experiments, 19-month old male mice with or without exercise for 4 weeks in the presence or absence of intraperitoneal injection of valeric acid (one injection per week) were subjected to surgery (panels **H**–**J**). **A**, **E**, **H** Training sessions of Barnes maze test. **B**, **F**, **I** Memory assessment of Barnes maze test. **C**, **D**, **G**, **J** Novel object recognition test. Results are mean ± S.D. with (panels **B** and **G**) or without (panels **A**, **E** and **H**) presentation of value of individual mouse or median ± interquartile range (panels **F**, **I**, and **J**) with presentation of value of individual mouse in other panels (*n* = 11–12 for panels **A**–**D**, = 9–10 for panels **E**–**G**, = 9–11 for panels **H**–**J**). Exe exercise, Sur surgery, NS normal saline, Val valeric acid. *P < 0.05 compared the two curves.

Since age is a risk factor for POCD [1, 2, 5], we determined whether those mechanisms that identified in the young adult mice operated in old mice for POCD. Nineteen-month old mice with surgery took longer than control mice to identify the target box in Barnes maze test. This increase was attenuated by exercise (Fig. 9A, B). Similarly, surgery impaired the performance of old mice in novel object recognition test and this impairment was attenuated by exercise (Fig. 9C, D). Surgery mice transplanted with feces from exercise mice performed better than surgery mice transplanted with feces from control mice in Barnes maze and novel object recognition tests (Fig. 9E–G). Also, intraperitoneal injection of valeric acid impaired the performance of surgery mice with exercise in Barnes maze and novel object recognition tests (Fig. 9H–J). These results suggest that exercise reduces the development of POCD via altering gut microbiota and that valeric acid may be a mediator for this effect in old mice.

**Fig. 10:**
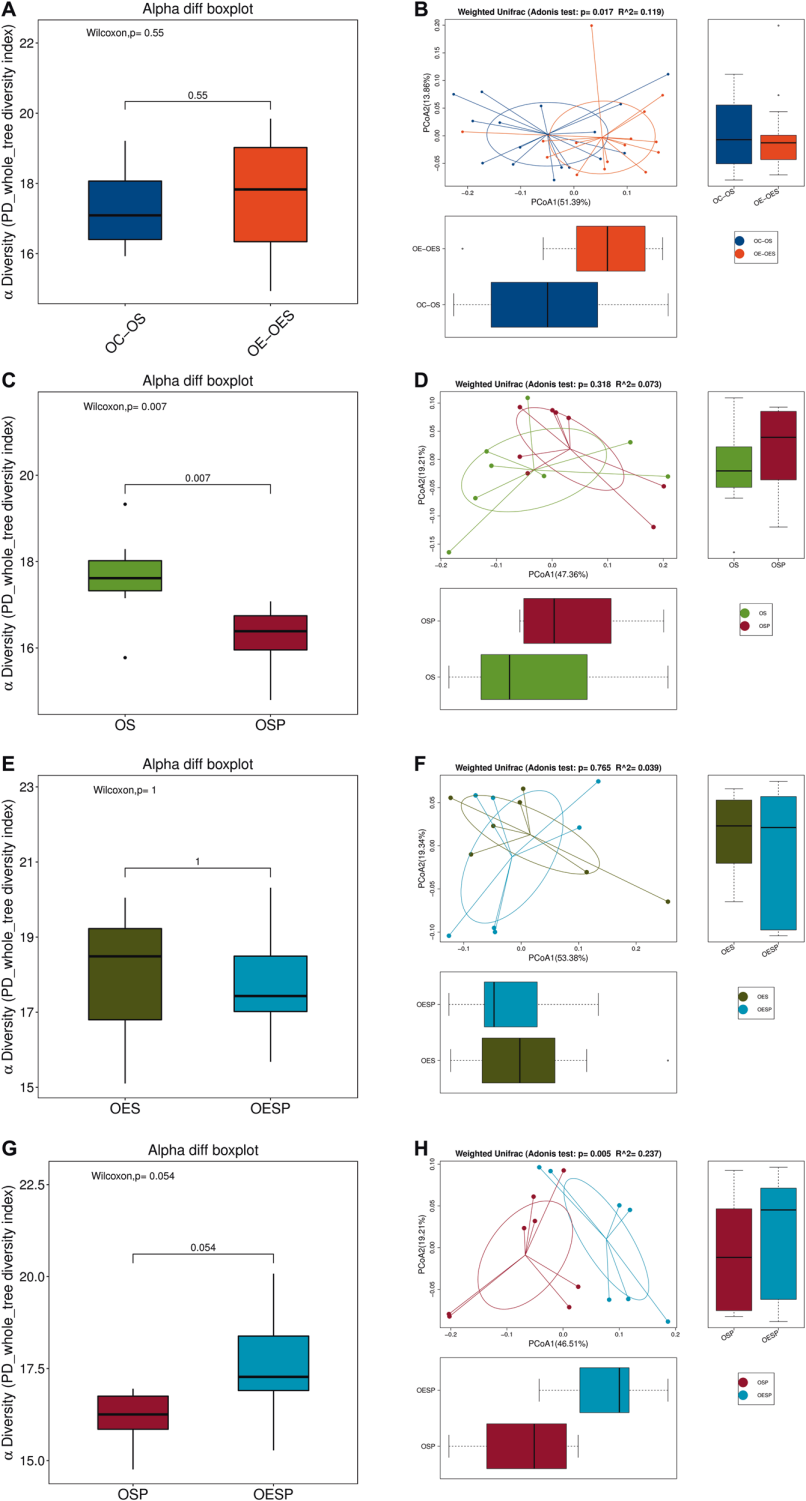
Exercise stabilized gut microbiota of mice with surgery in old mice. Nineteen-month old male mice with or without exercise for 4 weeks were subjected to left carotid artery exposure (surgery) under isoflurane anesthesia. Presentation of α diversity is in (panels **A**, **C**, **E** and **G**). Presentation of β diversity is in (panels **B**, **D**, **F** and **H**) (*n* = 16 for panels **A** and **B**, and = 8 for other panels). OC-OS old control mouse samples harvested before surgery, OE-OES old exercise mouse samples harvested before surgery, OS old surgery mouse samples harvested just before surgery, OSP old surgery mouse samples harvested on post-surgery day 7, OES old exercise mouse samples harvested before surgery, OESP old exercise mouse samples harvested on post-surgery day 7.

Exercise increased the β diversity among samples of old mice (Fig. 10A, B). Surgery decreased the diversity of gut microbiota in old control mice and this decrease was attenuated by exercise (Fig. 10C–H). Gut microbiota of exercise mice or surgery mice transplanted with feces from exercise mice had an abundant amount of *Bacteroidetes* and *Bacteroidales*, while gut microbiota of control mice or surgery mice transplanted with feces from control mice contained a large amount of *Clostridiales, Clostridia* and *Firmicutes* (Supplementary Fig. S12). These results suggest that exercise increases the diversity of gut microbiota and stabilizes the gut microbiota of surgery mice.

**Fig. 11:**
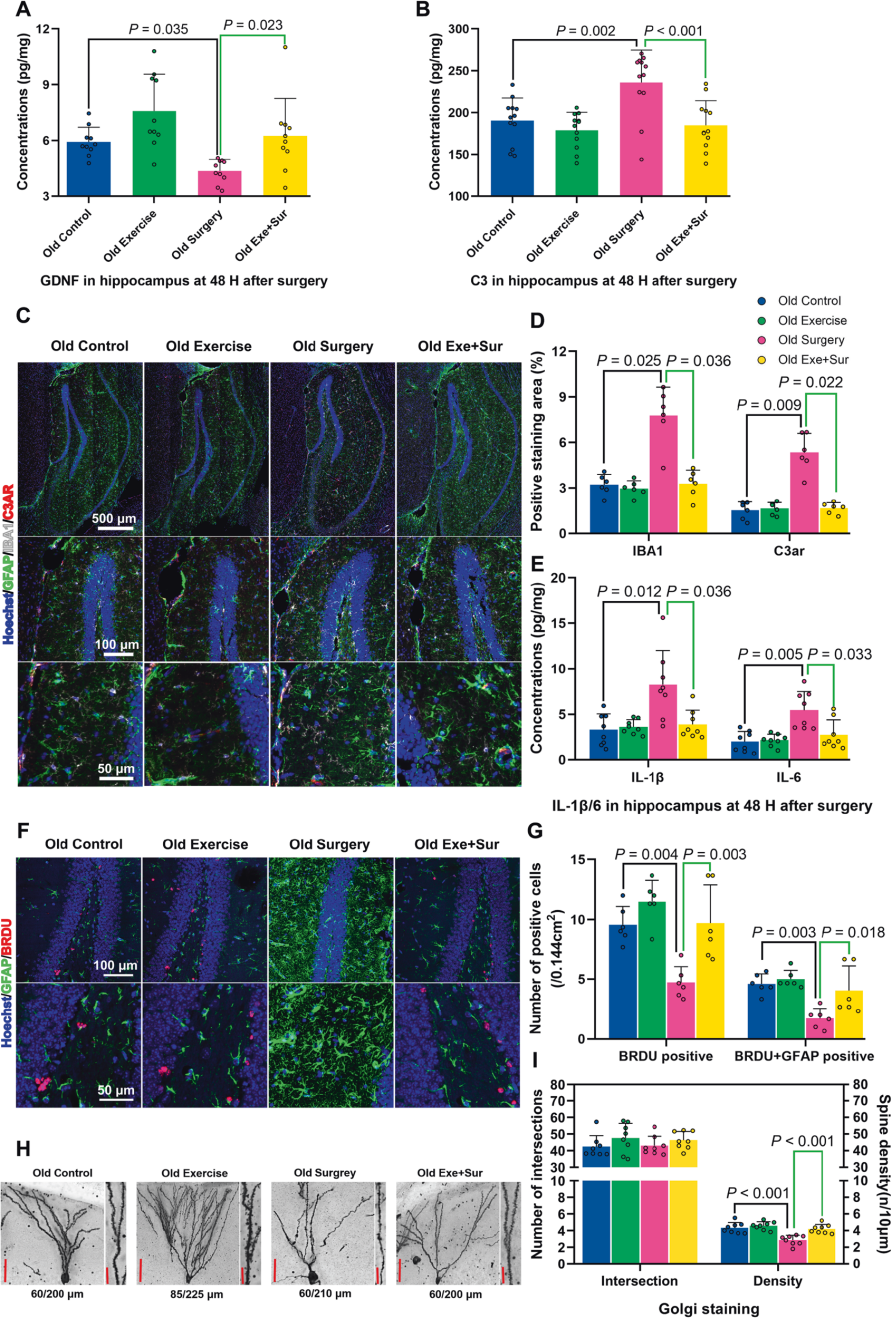
Exercise attenuated surgery-induced GDNF decrease, immune and inflammatory responses and dendritic arborization impairment in old mice. Nineteen-month old male mice with or without exercise for 4 weeks were subjected to left carotid artery exposure (surgery) under isoflurane anesthesia. **A** GDNF expression. **B** C3 expression. **C** Representative Iba-1 and C3ar immunostaining images. **D** Quantitative data of Iba-1 and C3ar immunostaining. **E** IL-1β and IL-6 expression. **F** Representative GFAP and BrdU immunostaining images. **G** Quantitative data of GFAP and BrdU immunostaining. **H** Representative Golgi staining. **I** Quantitative data of intersections among dendritic branches and spine density. Results in panels **A**, **D** and **E** are median ± interquartile range with presentation of value of individual mouse and results in panels **B**, **G** and **I** are mean ± S.D. with presentation of value of individual mouse (*n* = 10 for panels **A**, = 12 for panel **B**, = 6 for panels **D** and **G**, = 8 for panels **E** and **I**). Exe exercise, Sur surgery.

Although exercise did not alter valeric acid before surgery, exercise reduced valeric acid in surgery mice (Fig. 6C, E). Similar to the findings in young adult mice, surgery reduced GDNF in the hippocampus. This reduction was attenuated by exercise (Fig. 11A). Surgery increased the expression of C3, C3ar, Iba-1, IL-1β and IL-6. These increases were attenuated by exercise (Fig. 11B–E). In addition, surgery decreased brain cell generation and spine density and exercise reserved brain cell generation and spine density in surgery mice (Fig. 11F–I). Finally, surgery reduced the expression of PSD95 and synapsin-1. Exercise blocked this decrease (Supplementary Fig. S13). These results suggest that pathological processes similar to those in young adult mice occur in old mice after surgery and that these processes including neuroinflammation, decrease of growth factor expression and dendritic arborization impairment can also be inhibited by exercise.

## Discussion

Our results suggest that low intensity exercise attenuates surgery-induced learning and memory impairment as assessed by Barnes maze and novel object recognition tests. These beneficial effects occurred in both young adult and old mice. Physical activity ability has been associated with better preservation of mental status after surgery in humans [18]. The effects of exercise on POCD in general population have not been reported. Interestingly, our results showed that middle and high intensity exercise did not protect mice against learning and memory impairment after surgery, suggesting that only an appropriate level of exercise is protective. Consistent with this functional data, this level of exercise attenuated neuroinflammatory responses to surgery. Neuroinflammation is considered a critical pathological process for POCD [6, 7]. In addition, exercise attenuated surgery-induced decrease of brain cell genesis and dendritic arborization, structural bases for learning and memory [14, 22].

Previous studies have suggested an association between learning and memory dysfunction and gut dysbiosis after surgery [11–13]. Our studies showed that mice transplanted with feces from exercise mice had better performance in Barnes maze and novel object recognition tests than mice transplanted with feces from control mice after surgery. Transplanting feces from control mice or mice with surgery established their corresponding gut microbiota in control mice. The recipients of feces from surgery mice but not those receiving transplantation of feces from control mice developed learning and memory dysfunction compared with control mice without fecal transplantation. Mice transplanted with feces from exercise mice had decreased neuroinflammatory responses and increased brain cell genesis and dendritic arborization. In addition, surgery decreased gut microbiota diversity and exercise stabilized the gut microbiota after surgery. These results provide direct evidence that gut microbiota alteration after surgery may contribute to the learning and memory dysfunction after surgery and that exercise may work on gut microbiota to reduce neuroinflammatory responses and to preserve learning, memory, brain cell genesis and dendritic arborization.

Gut microbiota produces SCFAs that can affect various brain functions including learning and memory [27, 28]. However, previous studies often consider using a few SCFAs as a group for the effects [29–31]. Our results suggest the contribution of decreasing valeric acid to the effects of exercise because exercise reduced valeric acid in the blood under baseline condition and after surgery. Valeric acid was decreased in the blood of mice transplanted with feces of exercise mice. Although exercise altered the concentrations of other SCFAs, such as propionic acid, in young adult mice with surgery, valeric acid was the only SCFA whose concentration in the blood was decreased by exercise in old mice with surgery. In supporting the role of valeric acid in the exercise effects, intraperitoneal injection of valeric acid worsened the learning and memory of young adult and old mice with exercise and surgery. Valeric acid also increased the inflammatory responses and impaired brain cell genesis and dendritic arborization. Together, these results suggest a detrimental effect of valeric acid on the brain. However, valeric acid injected intracerebroventricularly at 1/50 dose injected intraperitoneally did not impair learning and memory. This dose was selected because the brain accounts for about 2% total body weight. The failure to induce learning and memory impairment by this approach may be expected because valeric acid can cross blood-brain barrier [27, 32]. Consistent with this idea, valeric acid was detected in the brain tissues and intraperitoneal injection of valeric acid increased valeric acid in the brain in our study. Thus, the small amount of valeric acid injected into the brain may cross blood-brain barrier to be redistributed to other tissues and organs, which decreases the concentrations of valeric acid in the brain. Additional high doses of valeric acid injected into the brain were not performed because it does not appear that this approach will provide clear-cut evidence on whether valeric acid works directly on the brain to have its effects on the brain. Of note, intraperitoneal injection of valeric acid did not increase valeric acid in the blood of mice with exercise and surgery. This result may be anticipated because the blood was harvested 7 days after the last injection of valeric acid (7 days after surgery). However, the amount of valeric acid in the cerebral cortex was increased 24 h after the last intraperitoneal injection of valeric acid, suggesting that these injections increase valeric acid in tissues and organs. Also, it is not uncommon that previous studies measure SCFAs in the feces in the investigation of determining their effects on various organs [33, 34]. Our results showed that the concentrations of SCFAs in the feces were much higher than those in the blood, consistent with a previous finding that only a small fraction of SCFAs in the feces can get into the circulation due to the gut absorption regulation and liver metabolism [28]. In addition, our study showed that surgery and exercise altered the concentrations of various SCFAs in the blood, they did not alter SCFAs in the feces. Thus, it is important to measure blood SCFAs when investigating their role in various organs.

Relatively limited information is known on how SCFAs affect cell activity and function. Recent studies suggest that SCFAs via working on their receptors or target proteins to activate intracellular signaling pathways, such as nuclear factor κB, to regulate the immune and inflammatory responses. SCFAs can also induce epigenetic regulation of gene expression including the expression of growth factors [27, 28]. Our results suggest that these downstream events may contribute to the effects of exercise and valeric acid on learning and memory after surgery. Exercise attenuated surgery-induced neuroinflammation and GDNF reduction. Valeric acid blocked these exercise effects. We have shown that GDNF may play a role in POCD in previous studies because surgery reduces GDNF and that intracerebroventricular injection of GDNF attenuates learning and memory dysfunction in rodents with surgery [6, 26]. Consistent with this GDNF role and the knowledge that neuroinflammation is critical for POCD [6, 7], we showed in this study that GDNF reduced neuroinflammation after surgery.

An important molecule downstream of valeric acid to induce the effect on learning and memory may be C3 signaling. Surgery increased C3 and C3ar. Exercise attenuated this increase. Intraperitoneal valeric acid blocked the effects of exercise on C3ar and C3 expression. Intraperitoneal valeric acid increased C3 in the brain of control mice. More importantly, the C3 antagonist, SB290157 [25], blocked surgery-induced learning and memory dysfunction. A C3 agonist worsened learning and memory of mice with exercise and surgery. These results suggest that C3 signaling is important for learning and memory dysfunction after surgery. Consistent with our findings, a just published study showed that old mice had increased C3 in the hippocampus after exploratory laparotomy and that compstatin, a C3 inhibitor, attenuated learning and memory dysfunction after the surgery [35]. Compstatin is a peptide inhibitor and its blood-brain barrier permeability is not known [24]. Compstatin was given systemically in the previous study [35]. It is unclear whether the effects of compstatin on the brain were through its systemic effect or working on the brain. We gave C3 agonist and antagonist directly into the brain, providing evidence to suggest the effects of C3 signaling in the brain on the learning and memory dysfunction after surgery. More importantly, our study has provided initial evidence to suggest that exercise and SCFAs, such as valeric acid, can regulate C3 signaling in the brain. Considering the broad effects of C3 signaling in the brain including regulating immune and inflammatory responses and neuronal activity [24], our study has identified a molecular mechanism for exercise to provide beneficial effects on the brain and for gut microbiota to affect the brain. In particular, our results suggest that exercise maintains astrocytes in an A2 phenotypic form because it reduced C3 and increased GDNF expression, a possible cellular mechanism for the beneficial effects of exercise.

We pooled feces from 7 to 10 donors to prepare fecal solution for transplantation as described previously [36]. This method does not capture well the effects of individual differences in gut microbiota [37] but shall provide excellent representations of gut microbiota of the specific condition to all recipients. Individual variation in recipient response shall be preserved in this approach. In addition, this method does not require arbitrary match between recipients and donors. The fecal solution was applied by gastric gavage and enema to the recipients. This method of application quickly and effectively established the transplanted gut microbiota in the recipients in our preliminary study, possibly because microbiota applied by enema will not be affected by acidic solution and solution containing various enzymes or chemicals in the upper gastrointestinal tract.

Our studies showed that exercise prior to surgery attenuated learning and memory impairment of mice with surgery. However, exercise did not affect the learning and memory of control mice. Exercise has been shown to improve learning and memory of animals modeled for various diseases [38, 39]. However, the effects of exercise on control animals are not consistent: both beneficial and no effects on learning and memory have been reported [40–42]. Specifically, four studies that used Barnes maze and/or novel object recognition tests to assess learning and memory in control mice with or without exercise were identified. One study showed that adult control rats with one episode of exercise that was at 60–70% maximal indirect oxygen uptake for 30 min immediately after the training session of novel object recognition test had better memory performance assessed 7 days but not at 1 day or 14 days after the training session [43]. The second study showed that voluntary exercise for 4 weeks did not affect the learning and memory assessed by Barnes maze and novel object recognition tests in control rats [44]. Two other studies had the data that swimming exercise improved memory of old rats assessed by novel object recognition test [45, 46]. A possible explanation for this inconsistency is that it requires appropriate levels of exercise to provide beneficial effects in control mice, as in the case shown in this study to prevent surgery-induced learning and memory dysfunction. This level of exercise was not specifically searched for because determining whether exercise improves learning and memory in control mice is not the focus of this study.

Our findings may have significant clinical implications. First, the study provided evidence to suggest that appropriate exercise levels are beneficial. It may not be difficult to understand that over-exhaustion may not be good for health. Since each individual has a different exercise capacity, it is wise to determine the maximal exercise capacity of the individual and have an individualized exercise protocol as we did in this study. This method shall be used in clinical situations if the beneficial effects of exercise are shown in humans. Second, our findings have suggested multiple potential interventions for reducing POCD, such as fecal transplantation and antagonizing C3 signaling. Third, our study suggests a detrimental role of valeric acid. Although there is no antagonist for valeric acid, these antagonists or methods to reduce blood valeric acid concentrations may be developed.

Our study has limitations. The possible detrimental effects of valeric acid on the brain are suggested by our study. However, we have not determined detailed mechanisms for valeric acid to induce these effects, other than providing evidence to suggest that C3 signaling activation may be a downstream event. Also, we focused on investigating the mechanisms for the beneficial effects of low intensity exercise but did not determine why higher intensity exercises lack beneficial effects on surgery-induced learning and memory dysfunction. Determining the mechanisms for this failure may provide insights on exercise physiology and pathophysiology and identify possible targets to be eliminated for the higher intensity exercises to be protective. However, it appears the need for this determination is not high since low intensity exercise is protective and easy to be achieved.

**Fig. 12:**
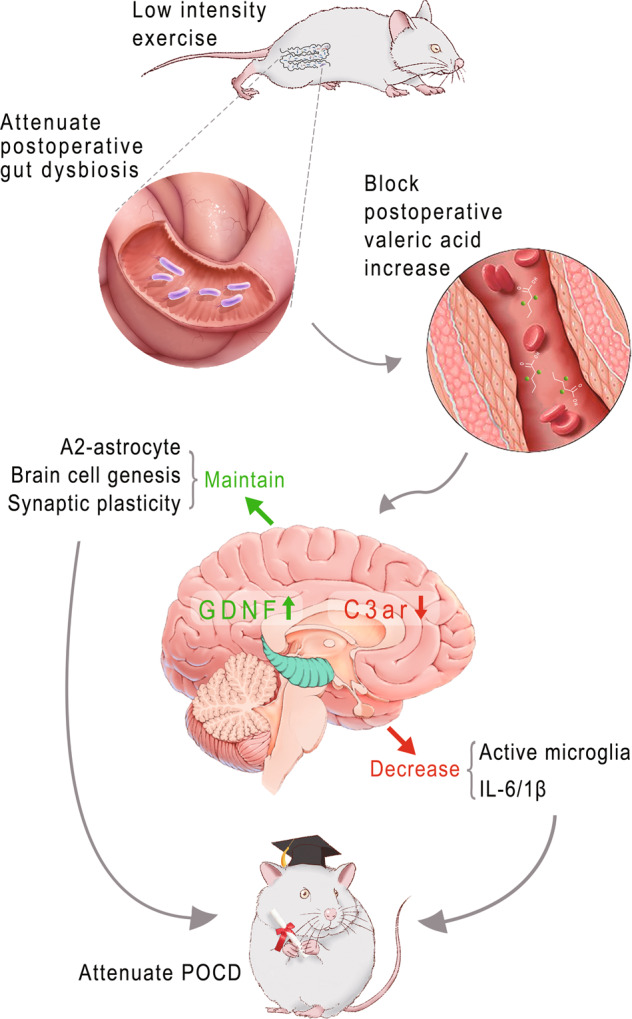
Diagrammatic presentation of the findings from this study. Surgery may induce gut dysbiosis to lead to increased valeric acid in the blood, which then activates complement 3 signaling to result in postoperative cognitive dysfunction. Low intensity exercise before surgery inhibits this detrimental pathway to reduce postoperative cognitive dysfunction. GDNF glial cell-derived neurotrophic factor, C3ar complement 3a receptor, POCD postoperative cognitive dysfunction.

In summary, our study suggests that low intensity exercise can stabilize gut microbiota after surgery. This effect reduces valeric acid in the blood, which then maintains the productions of growth factors, such as GDNF, and inhibits neuroinflammation (Fig. 12). Transplantation of healthy gut microbiota and antagonizing C3 signaling may be potential interventions to reduce learning and memory dysfunction after surgery.

## Materials and methods

The experimental protocols and procedures were approved by the Institutional Animal Care and Use Committee of the University of Virginia (Charlottesville, VA, USA; protocol number: 3114). All animal experiments were performed in accordance with the National Institutes of Health Guide for the Care and Use of Laboratory Animals (NIH publications number 80–23) revised in 2011.

The sources of key materials were listed in the Key Resources Table in the Supplementary Materials.

### Animals and experimental design

Eight-week old male C57BL/6 J mice weighing 19–22 g were housed in a room maintained under constant environmental conditions (temperature 22–24 °C, a 12 h light/dark cycle, and 50 ± 10% humidity) with free access to food and water. All of them were allowed to acclimate for one week before experiments. They were randomly assigned to the following groups in the first experiment: (1) control group (not being exposed to anesthesia and surgery), (2) exercise group (exercised at 35–40% maximal capacity, but not being exposed to anesthesia and surgery), (3) surgery group (left carotid artery exposure for 15 min under isoflurane anesthesia for 2 h), and (4) to (6) Exe-l+Sur, Exe-m+Sur and Exe-h+Sur groups: exercised at 35–40%, 55–60% and 75–80% maximal capacity, respectively, and were subjected to anesthesia and surgery. The animals were used for learning and memory tests starting 4 days after the surgery (*n* = 20) or their brains were harvested for biochemical assays at 6 h, 24 h, 48 h, 72 h, 96 h and 7 days after the surgery (*n* = 9 or 14), for immunofluorescent staining at 48 h and 19 days after the surgery (*n* = 6), and for Golgi staining at 19 days after the surgery (*n* = 8). Blood and feces from mice of the first 4 groups were harvested for measuring SCFAs at 7 days after the surgery (*n* = 7) and for 16 S analyses at 3 and 7 days after the surgery (*n* = 8) or at the end of 4-week exercise protocol (*n* = 8) or at the corresponding time in the first 2 groups (*n* = 16). Mice for tissue harvesting were different cohorts of mice that were used for learning and memory tests.

In the second experiment, mice were randomly assigned to: (1) Trans-control+Sur group and (2) Trans-exe+Sur group. Mice in the first group received 500 μl fecal solution from control group by gastric gavage and 600 μl by enema once a day for 7 consecutive days after antibiotic treatment to eliminate the native gut microbiota in the recipients. Mice in the second group received 500 μl fecal solution from exercise mice by gastric gavage and 600 μl by enema once a day for 7 consecutive days after the antibiotic treatment. Feces from the recipients were harvested 14 days after fecal transplantation for 16 S analysis. The mice were then subjected to surgery. Learning and memory were evaluated from 4 days after surgery (*n* = 15). Brain was harvested for biochemical studies as in the first experiment (*n* = 10).

In the third experiment, mice were randomly assigned to: (1) Trans-control group, and (2) Trans-control+Sur group. Mice in both groups received transplantation of feces from control mice. The second group had surgery 14 days after fecal transplantation. Hippocampus was harvested 2 days after surgery to measure GDNF (*n* = 10).

In the fourth experiment, mice were randomly assigned to: (1) control group (naïve mice that were not exposed to any experimental conditions described in this study), (2) antibiotic group that received antibiotics for 7 days, and (3) Trans-control group that received transplantation of feces from control mice. Learning and memory were assessed 19 days after the completion of fecal transplantation (26 days after the completion of antibiotic treatment in the antibiotic group) (*n* = 12).

In the fifth experiment, mice were randomly assigned to: (1) control group, (2) Trans-control group that received transplantation of feces from control mice, and (3) Trans-surgery group that received transplantation of feces from surgery mice. Fecal transplantation was performed as described for the second experiment. Feces from the recipients were harvested 14 days after fecal transplantation for 16 S analysis (*n* = 10). Learning and memory were assessed 4 days after the fecal sample was harvested (*n* = 17).

In the sixth experiment, mice were randomly assigned to: (1) surgery plus normal saline group that received 200 µl normal saline (NS) by intraperitoneal injection once a week for 4 weeks and then surgery, (2) exercise plus normal saline plus surgery group that received 200 µl NS by intraperitoneal injection once a week during the 4-week exercise at 35–40% maximal capacity and then surgery, (3) exercise plus valeric acid plus surgery group that received valeric acid (200 mg/kg in 200 µl) by intraperitoneal injection once a week during the 4-week exercise at 35–40% maximal capacity and then surgery. The injection was given in the morning of first exercise day of the week. One additional injection was performed on the surgery day. Behavioral (*n* = 13) and biochemical (*n* = 10) outcomes were assessed as in the second experiment.

In the seventh experiment, 13-week old (weighing 23–27 g) male C57BL/6 J mice were randomly assigned to: (1) control group, (2) NS group that received 4 µl NS by intracerebroventricular injection once daily for 4 consecutive days, and (3) valeric acid group that received 4 mg/kg in 4 µl by intracerebroventricular injection once daily for 4 consecutive days. The left and right cerebroventricles were injected on an alternated schedule. Learning and memory were assessed 4 days after the injection (*n* = 15).

In the eighth experiment, mice were randomly assigned to: (1) surgery plus dimethyl sulfoxide (DMSO) group that received DMSO by intracerebroventricular injection once daily for 4 consecutive days starting on the surgery day, (2) surgery plus C3ar antagonist group that received C3ar antagonist (SB290157) by intracerebroventricular injection once daily for 4 days starting on the surgery day, (3) exercise plus DMSO plus surgery group that received DMSO by intracerebroventricular injection once daily for 4 consecutive days starting on the surgery day after the 4-week exercise at 35–40% maximal capacity, and (4) exercise plus C3ar agonist plus surgery group that received a C3ar agonist by intracerebroventricular injection once daily for 4 consecutive days starting on the surgery day after the 4-week exercise at 35–40% maximal capacity. Behavior tests were started from 4 days after surgery as stated above (*n* = 15).

In the ninth experiment, mice were randomly assigned to: (1) control group, (2) surgery group, (3) surgery plus GDNF group that received GDNF by intracerebroventricular injection when they had surgery, and (4) surgery plus heat-inactivated GDNF group that received heat-inactivated GDNF by intracerebroventricular injection when they had surgery. Hippocampi were harvested at 48 h after surgery for ELISA study (*n* = 12).

After one-week acclimation, old mice (18-month old male C57BL/6 J mice) weighing 30–36 g were used in three studies. In the first experiment, old mice were randomly assigned to: (1) control group, (2) exercise group in which mice had 4-week exercise at 35–40% maximal capacity, (3) surgery group, and (4) Exe+Sur group in which mice had 4-week exercise at 35–40% maximal capacity before surgery. The animals were used for learning and memory tests starting 4 days after the surgery (*n* = 12) or their brains were harvested for biochemical assays at 48 h after the surgery (*n* = 10 or 12), for immunofluorescent staining at 48 h and 19 days after the surgery (*n* = 6), and for Golgi staining at 19 days after the surgery (*n* = 8). Blood and feces from mice of the 4 groups were harvested for SCFA measurement at 7 days after the surgery (*n* = 7) and for 16 S analyses at 3 and 7 days after the surgery (*n* = 8) or at the end of 4-week exercise protocol or at the corresponding time in the first 2 groups (*n* = 16). Mice for tissue harvesting were different cohorts that were used for learning and memory tests. In second experiment, old mice were randomly assigned to: (1) Trans-Old Control+Sur group, and (2) Trans-Old Exe+Sur group. These mice were subjected to surgery 14 days after fecal transplantation as described in the second experiment of young adult mice. Learning and memory were evaluated from 4 days after surgery (*n* = 10). In third experiment, old mice were randomly assigned to: (1) surgery plus NS group; (2) exercise plus NS plus surgery group; and (3) exercise plus valeric acid plus surgery group. These mice received exercise conditioning and valeric acid as described in sixth experiment of young adult mice. Behavioral outcomes were tested starting 4 days after surgery (*n* = 11).

The sample sizes (the n) described above represented the number of animals that were randomized into each group/condition. These numbers of animals were the sum of at least 3 replicates of each experiment. Animals were randomly distributed into groups based on computer-generated randomization tables in each experiment.

### Maximal exercise capacity determination and exercise training

Before surgery, 8-week and 18-month old mice were exposed to a 4-week treadmill aerobic exercise after a determination of maximal exercise capacity of individual mouse. This determination was performed in a way similar to those described before [47, 48]. Briefly, the mice were acclimated to the treadmill in the first day with initial settings of shock grid at 25 V, 0.3 mA, and 2 Hz and the speed and inclination at zero for 10 min. The treadmill speed was then increased to 10 cm/s with inclination set to 5^0^ for 10 min. Next, the treadmill speed and inclination were increased to 15 cm/s and 10^0^ for 5 min. The speed was then increased by 5 cm/s every 5 min to an average maximal speed of 70 cm/s in young mice and 60 cm/s in old mice (around 65–75 cm/s and 55–65 cm/s, respectively), as they reached to the criteria for exercise-induced exhaustion. These criteria were: (1) 10 consecutive seconds on the electric grid; (2) spending >50% of time on the grid; and/or (3) lack of motivation to manual prodding. The mouse was removed immediately from the respective lane once one or more of these criteria was met. On the next day, mice ran on the same treadmill at half of average maximal speed (35 cm/s in young mice or 30 cm/s in old mice) and 10^0^ of inclination until they reached one of the exhaustion criteria again, and the duration of continuous exercise was recorded as the maximal exercise capacity of each mouse. After these two protocols, mice were housed separately for 30 min to avoid noticeable aggressive behavior following exercise. After resting for 2 days, mice assigned to exercise training groups were subjected to a 4-week protocol of forced treadmill running at 35–40%, 55–60% or 75–80% of the maximal capacity, respectively, for 5 days a week. Mice that were shocked for >5% of the total daily exercise time in 2 consecutive days or >3% of the total time in 3 consecutive days would be excluded. The non-exercise groups in the same set of experiments with the exercise groups were placed on a non-moving treadmill daily for about 30–40 min. They received 10 shocks for a total of 5 s, the average amount of shocks that mice in an exercise group received each day, during their stay in non-moving treadmill. If a set of experiments did not have an exercise group, no mice in the set of experiments received shocks.

### Antibiotic treatment

To facilitate the colonization of the transplanted microbiota, recipient mice were given an antibiotic treatment once daily for 7 consecutive days to eliminate their original gut microbiota as described by us and others [11, 36]. In brief, after purgation with 10% magnesium sulfate (200 μl/10 g, twice with 3 h interval) by gastric gavage in the first day, C57BL/6 J mice received amoxicillin/clavulanic acid (200 mg/kg), metronidazole (200 mg/kg) and cefazolin (2 g/kg) by gastric gavage and enema (in 300 μl for gastric gavage and 400 μl NS for enema) in the morning once a day for 7 consecutive days, and amoxicillin/clavulanic acid (20 mg/kg), metronidazole (20 mg/kg) and cefazolin (300 mg/kg) in 200 μl NS daily by intraperitoneal injection for the same 7 days (at 4 pm to 5 pm). To prevent bacterial cross contamination, mouse handling and daily cage and water bottle changes were performed by technicians wearing a clean gown and gloves in a ventilated hood. Feces from these mice were harvested 24 h after the last treatment for 16 S analyses to determine the effect.

### Fecal transplantation

Fresh fecal pellets collected within 30 min after bowel movement from control mice, mice immediately after the completion of 4-week exercise or mice 2 to 8 days after surgery were diluted with 100 mg/ml sterile saline. All fecal pellets of 7–10 donors with each experimental condition were mixed and re-suspended together in saline. Briefly, the fecal matter was vortex-mixed for 5 min and then passed through 70 μm nylon cell strainer to remove undigested food and particulate materials. The recipient mice received 500 μl corresponding fecal solution by gastric gavage in the morning and 600 μl by enema in the afternoon from 24 h after the last dose of antibiotics for 7 consecutive days. Transplanted mice were maintained for 2 weeks after fecal transplantation before they were subjected to fecal collection for 16 S analyses and surgery.

### Anesthesia and surgery

The surgery was left carotid artery exposure as we described before [6]. Briefly, mice were anesthetized by 2% isoflurane, and kept spontaneous respirations with a facemask supplied with 100% oxygen during the procedure. Rectal temperature was monitored and maintained at 37 °C with the aid of a heating blanket. A 2-cm midline neck incision and soft tissue dissection with 1-cm long common artery exposure were performed without any damage to the vagus nerve after the mouse was anesthetized by isoflurane for at least 20 min. The wound was then irrigated and closed by using 4–0 surgical suture. The surgical procedure was performed under sterile conditions and lasted around 15 min in young mice and 12 min in old mice. The total duration of general anesthesia was 2 h. After the surgery, all animals received a subcutaneous injection of 3 mg/kg bupivacaine. No response to toe pinching was observed during the anesthesia.

### Intracerebroventricular injections

As described above, some groups of mice received intracerebroventricular injection. Briefly, these mice were placed in a stereotactic head frame in the prone position. The injection site was located as: 1.00 mm mediolateral, −0.3 mm anteroposterior from Bregma, and −2.5 mm dorsoventral depth. Mice in surgery plus GDNF group received intracerebroventricular injection of 10 μg/kg recombinant mouse GDNF in 3 μl phosphate-buffered saline (PBS) as described in previous studies [6, 26], and mice in surgery plus heat-inactivated GDNF group received injection of heat-denatured (5 min at 100^0 ^C) GDNF solution. Mice in surgery plus C3ar antagonist group received 10 μg/kg SB290157 in 3 μl PBS containing 5% DMSO at 0 h, 24 h, 48 h and 72 h after surgery. Mice in exercise plus C3ar agonist and surgery group received 1 μg/kg C3ar agonist in 3 μl PBS containing 5% DMSO at the same time as for SB290157. Mice in valeric acid group of the sixth experiment received 4 mg/kg valeric acid in 4 µl for 4 consecutive days.

### Behavioral testing

Learning and memory were evaluated by novel object recognition and Barnes maze test. All behavioral tests were conducted at 10:00 am−5:00 pm in a sound-isolated room. Mice used in the learning and memory tests were not used for any biochemistry studies to avoid the effects from these tests.

### Novel object recognition test

As we and others described before [20, 49, 50], mice were put in an open-field chamber for 5 min for habituation 4 days after surgery. The test was performed in the following way. Two of the same objects were placed at adjacent angles of the chamber on the learning day. Mice were put into the chamber with their backs turned towards the objects and allowed to explore the chamber freely for 5 min. The animal was eliminated if the total exploration time on two objects was <5 s. One of the objects was replaced by a novel object 30 s or 24 h later. The mouse was put into the chamber with their backs turned towards the objects and allowed to explore for 5 min. Animal behavior was recorded by ANY-maze behavioral tracking software (Stoelting Co., IL). Exploratory time of new (T2) and old (T1) objects within 5 min was recorded and the memorization ability of the mouse was quantified by discrimination index: DI = T2 / (T1 + T2). The DIs at 30 s and 24 h after the training reflected the instant and long-term memory, respectively. The field was always provided with even light, and the objects and fields were cleaned with 70% ethanol after each test.

### Barnes maze

Seven days after surgery, animals were subjected to Barnes maze to test their spatial learning and memory as we previously described [6, 49]. Barnes maze is a circular platform with 20 equally spaced holes (SD Instruments, San Diego, CA). One of the holes was connected to a dark chamber that was called target box. The test started by placing animals in the middle of the Barnes maze. Aversive noise (85 dB) and bright light (200 W) shed on the platform were used to encourage mice to find this box. After training for 4 days, their reference memory was tested on day 5 and day 12. No test was performed during the period from day 5 to day 12. The latency to enter the target box during each trial was recorded by an ANY-Maze video tracking system (SD Instruments).

### Gut microbiota profiling

Fresh feces from mice were collected for gut microbiota profiling. Mice were placed individually in an autoclaved cage for collecting feces and allowed to defecate freely. Feces were collected immediately in the sterile Eppendorf tubes on dry-ice and then stored at −80 °C until further processing. Bacteria DNA was extracted with Power Lyzer Power soil DNA isolation kit according to our previous protocols [11]. Sequencing libraries of the hypervariable V3–V4 region were prepared according to the Illumina MiSeq system instructions. Briefly, 12.5 ng DNA was used as DNA template for the first 16 S rRNA PCR. Primers used were as follows: F: 5′-TCGTCGGCAGCGTCAGATGTGTATAAGAGACAGCCTACGGGNGGCWGCAG-3′ and R: 5′-GTCTCGTGGGCTCGGAGATGTGTATAAGAGACAGGACTACHVGGGTATCTAATCC-3′. The amplicons were then cleaned up with AMPure XP magnetic beads and then used for Index PCR by using the Nextera XT Index Kit. Qubit dsDNA HS assay kit and TapeStation high sensitivity D1000 ScreenTape (Agilent, Blacksburg, VA, USA) were used to measure the concentrations of PCR products and normalize the quantity for library preparation. Sequencing was operated on an Illumina MiSeq instrument by MiSeq reagent kit v2 (500 cycles). Data was analyzed with the MiSeq Reporter software Metagenomics workflow v2.5.1.3 (Illumina, San Diego, CA, USA).

The paired reads obtained by double-terminal sequencing were spliced into a sequence through Pandaseq software. The long reads with high variable region were obtained. The reads whose average phred score in the window (5 bp in size, 1 bp step length) was less than 20 were trimmed. Reads containing ambiguous “N” or with length < 220 bp were discarded. After quality control of the original data, the high-quality sequences without chimeras were arranged according to abundance from large to small and then clustered with 97% similarity into an Operational Taxonomic Units (OTU). Each OTU was considered to represent a species. To avoid the deviation of analysis caused by the different sizes of sample sequencing data, the number of Reads to OTU was entered according to the minimum sequence number matched to OTU when the sequencing depth was sufficient. Alpha diversity was analyzed by random leveling. A Read was extracted from each OTU as a representative sequence. The representative sequence was compared with ribosomal database project database. Species were classified for each OTU, and species abundance tables were obtained for subsequent analysis. OTU picking was performed using Uclust on the software platform QIIME v1.9.1. Alpha diversity including Chao1 index and goods coverage index was used for the analysis of species diversity in a sample. Beta diversity analysis was used to compare differences in species diversity among samples and characterized by principal coordinate analysis (PCoA) based on Bray–Curtis distance. The differences among the groups were tested by analysis of similarity (ANOSIM).

### Plasma SCFA profiling

Plasma SCFA concentrations were quantified using a 7890–5977 gas chromatography-mass spectrometry (GC-MS, Agilent, Blacksburg, VA, USA). Plasma samples were collected from mice at time points described above, stored at −80 °C quickly and then thawed to room temperature for processing. Briefly, 100 μl plasma sample was vortex-mixed with 900 μl ethanol (containing 0.5% HCl, V/V). Ultrasonic treatment was applied for 40 min and then the samples were centrifuged at 14,000 RPM for 10 min. The supernatant of samples were measured by GC-MS analysis on an Agilent 7890–5977 GC-MS with electron impact ionization and a DB-FFAP capillary column (30 m × 0.25 mm × 0.25 μm).

### Cortical valeric acid quantification

The amount of valeric acid in the cerebral cortex harvested 24 h after the last intraperitoneal injection of valeric acid was determined by a method similar to that reported before [51, 52]. Briefly, 0.1 g cerebral cortex was homogenized in 500 µl aqueous acetonitrile. Supernatant was extracted with 8 ml extraction buffer (hexane:diethyl ether = 1:1) and centrifuged at 1800 g for 5 min. 7.5 ml supernatant was collected, mixed with 93 µl 20 mM KOH in methanol and dried at 40 °C under nitrogen gas. Dried residue was reconstituted in 50 µl 2.5% 18-Crown-6 in acetonitrile and was further derivatized with 9-chloromethylanthracene in acetonitrile with addition of tetramethylammonium hydroxide. Finally, 30 µl derivatization solution was loaded to Acclaim C18 column (3 µm, 4.6 × 100 mm, Thermo Scientific) and separated in Ultimate 3000 high performance liquid chromatograph (HPLC, Thermo Scientific) equipped with UV-visible detector. The peak of derivatized valeric acid was detected at a wavelength of 254 nm. 2-Ethylbutyric acid (2-EA) was added as an internal reference control. The peak area of valeric acid was measured as mAU*min and its peak area of each sample was normalized with that of 2-EA in the sample. Final results were presented as ratios of valeric acid group to NS group.

### Brain tissue harvesting

Mice were weighed and anesthetized with isoflurane at 6 h, 24 h, 48 h, 72 h, 96 h and 7 days after surgery and perfused with 4 °C NS for brain tissue harvesting. These hippocampi were used for ELISA analysis or RNA assay. Whole brain at Bregma −3 to −6 mm was used for immunofluorescent staining. All dissection procedures were performed on ice.

### mRNA library preparation and illumina Hiseq sequencing

The hippocampi harvested at 24 h after surgery from control group, surgery group, and Exe+Sur group in the first experiment were placed in liquid nitrogen for 1 h, and then stored in −80 °C freezer quickly for no >1 week. RNA purification, reverse transcription, library construction and sequencing were performed at WuXi NextCODE in Shanghai according to the manufacturer’s instructions (Illumina). The mRNA-focused sequencing libraries from total RNA were prepared using Illumina TruSeq^®^ RNA sample preparation Kit. PolyA mRNA was purified from total RNA using oligo-dT-attached magnetic beads and then fragmented by fragmentation buffer. Taking these short fragments as templates, first strand cDNA was synthesized using reverse transcriptase and random primers, followed by second strand cDNA synthesis. Then the synthesized cDNA was subjected to end-repair, phosphorylation and ‘A’ base addition according to Illumina’s library construction protocol. Illumina sequencing adapters were added to the cDNA fragments. After PCR amplification for DNA enrichment, the AMPure XP Beads (Beckmen) were used to clean up the target fragments of 200–300 bp.

After library construction, Qubit 3.0 fluorometer dsDNA HS Assay (Thermo Fisher Scientific) was used to quantify concentrations of the resulting sequencing libraries, while the size distribution was analyzed using Agilent BioAnalyzer 2100 (Agilent).

Sequencing was performed using an Illumina system following Illumina-provided protocols for 2 × 150 paired-end sequencing in WuXi NextCODE in Shanghai, China.

### Hippocampal mRNA expression by quantitative polymerase chain reaction (q-PCR)

The hippocampus harvested at 24 h after surgery from the control group, exercise group, surgery group, Exe+Sur group, Trans-Control+Sur group, Trans-Exe+Sur group, Exe+Sur+NS group, and Exe+Sur+Val group was placed in liquid nitrogen for 1 h, and then stored in −80 °C freezer for no more than 3 days. RNA extraction, RNA-to-cDNA reverse transcription, and q-PCR were performed as we previously described [49, 53]. Briefly, RNA was extracted by RNeasy Micro kit. Reverse transcription was finished by using 5X All in-One MasterMix. Finally, CFX connect real time system (Bio-Rad) was used for quantitative quantification. Primers for qPCR were: C3ar1-F: TGGGCTGGTGCTGTGGGTAG and C3ar1-R: GATGGCGAAGGCGGTTCTCAC. Relative gene expression for C3ar1 was calculated using the comparative threshold cycle ΔΔCt and housekeeping gene β-actin for normalization of gene expression. The mRNA levels for target genes are expressed as fold increases relative to relevant group, respectively.

### Cytokines and GDNF determination in hippocampus

IL-1β, IL-6, C3 and GDNF in the hippocampus were detected by using ELISA kits. For testing the IL-1β, IL-6 and GDNF, brain tissues were homogenized on ice in the RIPA buffer containing 25 mM Tris-HCl with pH 7.6, 150 mM NaCl, 1% sodium deoxycholate, 0.1% SDS and a protease inhibitor cocktail containing 10 mg/ml aproteinin, 5 mg/ml peptastin, 5 mg/ml leupetin and 1 mM phenylmethane sulfonylfluoride. For C3 testing, brain tissues were homogenized on ice in the PTR buffer (supplied in the kit). The supernatant was collected for ELISA detection after the homogenate was centrifuged at 13,000 g for 20 min at 4 °C and the protein concentration was determined by the BCA protein assay (Bio-Rad, Hemel Hempstead, Herts, UK). IL-1β, IL-6, C3 and GDNF in supernatant of brain were detected according to the manufacturer’s instruction for ELISA, and the amount of them in each sample in the brain was normalized by its total protein content and was expressed as pg/mg protein.

### Western blotting

Western blotting was performed as previously described [49]. In brief, protein concentrations of samples were determined using the BCA protein assay. Twenty micrograms of each sample were subjected to Western blotting analysis using the following primary antibodies: rabbit polyclonal anti-postsynaptic density protein 95 (PSD95) at 1:1000 dilution, rabbit polyclonal anti-synapsin 1 at 1:1000 dilution and rabbit anti-α-tubulin antibody at 1:1000 dilution. Images were scanned by an Image Master II scanner (GE Healthcare, Milwaukee, WI, USA) and analyzed using ImageQuant TL software v2003.03 (GE Healthcare). The band signals of interested proteins were normalized to those of the corresponding α- tubulin and expressed as fractions of control sample from the same gels.

### Immunofluorescent staining

The immunofluorescent labeling and quantification of the staining were performed as we have described before [6, 54]. Briefly, brain was fixed in 4% paraformaldehyde at 4 °C for 24 h and then incubated in 30% sucrose over night at 4 °C before being frozen in optimal cutting temperature compound. Coronal 20-µm thick sections were cut sequentially from Bregma −3 to −6 mm by using a cryostat and mounted on microscope slides. After being washed in Tris-buffered saline (TBS), sections were blocked in 10% donkey serum plus 1% bovine serum albumin (BSA) in TBS containing 0.3% triton-X 100 for 2 h at room temperature and then incubated at 4 °C overnight with the following primary antibodies: rabbit monoclonal anti-C3 antibody (1:50), goat monoclonal anti-GFAP antibody (1:200), rat monoclonal anti-C3ar antibody (1:50) and rabbit monoclonal anti-Iba-1 antibody (1:200). Sections were rinsed in TBS with 0.1% Triton-x 100. The donkey anti-rabbit IgG antibody conjugated with Alexa Fluor 594 (1:200), donkey anti-goat IgG antibody conjugated with Alexa Fluor 488 (1:200), donkey anti-rat IgG antibody conjugated with Alexa Fluor 594 (1:200) or donkey anti-rabbit IgG antibody conjugated with Alexa Fluor 647 (1:200) were incubated with the sections for 1 h at room temperature in the dark. The sections were washed in TBS, incubated with Hoechst 33342 (1:1000) for nuclear staining, rinsed and mounted with Vectashield mounting medium (H-1000; Vector Labs, Burlingame, CA). Images of immunostaining were acquired by z-stack with an LSM710 microscopy system (ZEISS), and a negative control omitting the incubation with the primary antibody was included in all experiments. The quantification was performed as described previously [6, 54]. Briefly, the whole dentate gyrus (DG) region that was covered by 2–3 non-overlapping fields from each of six sequential hippocampus sections of one mouse was imaged. The number of pixels per image with intensity above a predetermined threshold level was considered as a positively stained area for an interested marker and quantified using the Image-pro plus 6.0 (Media Cybernetics, Inc., Rockville, MD, USA) and presented as percentage of positive area in the total area. Six measurements per mouse were then averaged to reflect the level of positive staining. All quantitative analyses were performed in a blinded manner.

### BrdU administration and immunofluorescent staining

Seven days after surgery, mice were given 7 consecutive intraperitoneal injections of 80 mg/kg 5′-bromo-2′- deoxyuridine (BrdU) at 12:00 once daily as previously described [14]. Mice were sacrificed 5 days later for harvesting hippocampus. Hippocampus was fixed in 4% paraformaldehyde in 0.1 M phosphate-buffered saline at 4 °C for 24 h and embedded in paraffin. Five-micron thick coronal brain sections were cut sequentially from Bregma −2 to −4 mm. Antigen retrieval was performed by incubating sections with sodium citrate buffer containing 10 mM sodium citrate, 0.05% Tween 20 (pH 6.0) at 95–100 °C for 20 min. DNA denaturation was done by incubating with 1 N HCl on ice for 3 min, 2 N HCL at room temperature for 3 min, and at 37 °C for 6 min. Sections were blocked with 5% donkey serum in phosphate-buffered saline (PBS) containing 0.5% triton-X 100 for 2 h at room temperature. The sections were then incubated overnight at 4 °C with the following primary antibodies: rat monoclonal anti-BrdU antibody (1:100), and goat monoclonal anti-GFAP antibody (1:200). The sections were incubated with donkey anti-rat IgG antibody conjugated with Alexa Fluor 594 (1:200) or donkey anti-goat IgG antibody conjugated with Alexa Fluor 488 (1:200) for 1 h at room temperature in a dark room. After being washed in PBS, sections were counterstained with Hoechst 33342 (1:1000), rinsed and mounted with Vectashield mounting medium (Vector Labs). Images were acquired with a fluorescence microscope with a charge-coupled device camera (High Mag Olympus BX51) and a LSM710 confocal microscopy system (ZEISS). A negative control omitting the incubation with the primary antibody was included in all experiments. For each mouse brain, six sequential hippocampal sections were used for cell counting. The number of all cells positively stained for an interested marker or the combination of two markers in the sub-granular zone of the DG of each section was counted. The quantitative analyses were performed in a blinded manner.

### Golgi stain

Golgi staining was performed using FD Rapid GolgiStainTM Kit. Nineteen days after surgery, brains of 6 mice in each group were immersed in the impregnation solution (solutions A and B: 1:1) for 2 weeks and then transferred to solution C for 3 days. Coronal brain sections at a thickness of 100 μm and around −2.7 mm from bregma were cut on a vibratome (Microslicer^®^ 10110, Ted Pella, Inc. California, USA). As described before [55, 56], more than 10 well individualized neurons in the DG region of the hippocampus were randomly selected from each mouse, and sequential optical multiple-layers scanning images were taken at an interval 2.0 μm along the *z* axis (3DHISTECH, Pannoramic MIDI, Hungary) with 20 × objective. The multiple-layer scanning files were superposed to become one clear scanning file that can be viewed with Caseviewer (3DHISTECH, Hungary) on a computer. The total branch number and dendritic length were measured by Fiji software (Fiji-win64, NIH, USA). The complexity of total dendritic trees (intersections) was estimated using Sholl analysis. For spine density measurement, 5 neurons were selected from each animal. Five randomly selected microscopic fields at the apical or basal dendrites were photographed from the scanning files. The spine numbers in 40 μm segments were counted by an observer who was blind to group assignment. The results were expressed as the number of spines/10 μm segments. The data of dendritic branch numbers, length and intersections as well as spine density from one mouse were averaged to reflect the level of the mouse.

### Statistical analysis

Sample size calculation for each experiment was not performed. The determination of sample size was mostly based on our experience: larger sample sizes were used for experiments testing learning and memory than those for biochemical studies. All data that were collected were included in analyses. Missing data included the animals that met pre-determined exclusion criteria for exercise training and animals that died before the intended observation period. The number of animals that contributed data for analysis for each experimental condition is stated in figure legends. Results are presented as means ± S.D. (normal distribution data) or median ± interquartile range (not normal distribution data) with the presentation of individual animal data in the bar graph and means ± S.D. in the line plots. Data from the training sessions of Barnes Maze were analyzed by two-way repeated analysis of variance (ANOVA) followed by Tukey’s test. Gut microbiota data were analyzed as described in section Gut microbiota profiling. The other data were analyzed by one-way ANOVA followed by Tukey test if the data were normally distributed, by one-way ANOVA on rank followed by Tukey test or Student-Newman–Keuls Method if the data were not normally distributed, by two-way ANOVA, by *t* test or rank sum test. Significant difference was defined as *P* < 0.05 based on two-tailed hypothesis testing. Adjustments for multiple comparisons were not performed. All statistical analyses were performed with SigmaStat (Systat Software, Inc., Point Richmond, CA, USA).

## Supplementary information

Supplemental materials

## Data Availability

The RNA-seq data are at https://figshare.com/articles/dataset/Appropriate_exercise_level-gut_dysbiosis-neuroplasticity_and_cognitive_function_after_surgery_in_mice/15078294? Other data will be available upon reasonable request.
