# Supplementary material for: Appropriate exercise level attenuates gut dysbiosis and valeric acid increase to improve neuroplasticity and cognitive function after surgery in mice
Source: Mol Psychiatry. 2021 Oct 18;26(12):7167–87. doi: 10.1038/s41380-021-01291-y (PMC8873004; doi:10.1038/s41380-021-01291-y)
Supplement: Supplementary file 1 — Supplemental materials [file 41380_2021_1291_MOESM1_ESM.pdf]

Appropriate exercise level attenuates gut dysbiosis and valeric acid increase to improve neuroplasticity and cognitive function after surgery in mice

Zhongmeng Lai, Weiran Shan, Jun Li, Jia Min, Xianzhang Zeng, Zhiyi Zuo

KEY RESOURCES TABLE

REAGENT or RESOURCE	RESOURCE	IDENTIFIER
**Antibodies**		
Rabbit polyclonal anti-PSD95	Abcam	Cat#GR326885-1
Rabbit polyclonal anti- Synapsin 1	Abcam	Cat#GR3275672-2
alpha-Tubulin rabbit Ab	cell signaling Technology	Cat#2144S
Rabbit monoclonal anti-C3 antibody	Abcam	Cat#GR3273564-7
Goat monoclonal anti-GFAP antibody	Millipore	Cat#GR3313543-1
Rat monoclonal anti-C3ar antibody	Hycult Biotech	Cat#HM1123-27428M0819-A
Rabbit monoclonal anti-Iba-1 antibody	Wako Chemical Co.	Cat#019-19741
Rat monoclonal anti-BrdU antibody	Abcam	Cat#GR3289293-1
Donkey anti-goat IgG antibody conjugated with Alexa Fluor 488	Invitrogen	Cat#1463163
Donkey anti-rabbit IgG antibody conjugated with Alexa Fluor 594	Invitrogen	Cat#1987293
Donkey anti-rat IgG antibody conjugated with Alexa Fluor 594	Invitrogen	Cat#1979379
Donkey anti-rabbit IgG antibody conjugated with Alexa Fluor 647	Invitrogen	Cat#1826679?
Hoechst 33342	Thermo Scientific	Cat#U12842162
**Drugs**		
Amoxicillin/Clavulanic acid	Sigma-Aldrich	Cat#SM800607
Metronidazole	Sigma-Aldrich	Cat#M3761
Cefazolin	West-Ward	Cat#319033.1

Valeric sodium	Toronto Research Chemicals	Cat#V091420
**Critical Commercial Assays**		
Nylon cell strainer	Fisher Scientific	Cat#08-771-2
RNeasy Micro kit	QIAGEN	Cat#74004
5X All in-One MasterMix	Applied Biological Materials	Cat#G485
NovaSeq 6000 S4 Reagent kit	Illumina	Cat#20012866
DNA isolation kit	QIAGEN	Cat#12855-100
AMPure XP magnetic beads	Beckman Coulter	Cat#A63881
Nextera XT Index Kit	Illumina	Cat#FC-131-2001
Qubit dsDNA HS assay kit	Thermo Scientific	Cat#Q33230
FD Rapid GolgiStain kit	FD NeuroTechnologies	Cat#PK401
Mouse IL-1β ELISA kits	R&D SYSTEM	Cat#SMLB00C
Mouse IL-6 ELISA kits	R&D SYSTEM	Cat#SM6000B
Mouse C3 ELISA kits	Abcam	Cat#ab263884
Mouse GDNF ELISA kits	Biosensis	Cat#BEK-2229-2P
**Chemicals**		
Recombinant mouse GDNF	Abcam	Cat#GR2862-22
C3ar-agonist	cayman chemical	Cat#21683
C3ar-antagonist	Sigma-Aldrich	Cat#559410
BrdU	Sigma-Aldrich	Cat#APN17133-1-1
9-chloromethylanthracene	TCI America	Cat#: A5502
18-Crown-6	Sigma-Aldrich	Cat#: 80833
2-Ethylbutyric acid	Millipore Sigma	Cat# 109959
10×RIPA buffer	Thermo Scientific	Cat#89901
Protease inhibitor cocktail	Sigma-Aldrich	Cat#SRE0055
Tetramethylammonium hydroxide	TCI America	Cat# T0138

Figure legends for supplemental figures

Fig. S1. *Exercise altered gut microbiota.* Nine-week old male mice were with or without exercise for 4 weeks. Presentation of α diversity is in panels A and B. Presentation of β diversity is in panel C. Bacterial abundance is presented in panel D. n = 16 for all panels. C-S: control mouse samples harvested before surgery, E-ES: exercise mouse samples harvested before surgery.

Fig. S2. *Exercise stabilized gut microbiota after surgery.* Nine-week old male mice with or without exercise for 4 weeks were subjected to left carotid artery exposure (surgery) under isoflurane anesthesia. Presentation of α diversity is in panels A to D. Presentation of β diversity is in panels E and F. n = 8 for all panels. S: surgery mouse samples harvested just before surgery, SP3: surgery mouse samples harvested on post-surgery day 3, SP7: surgery mouse samples harvested on post-surgery day 7, ES: exercise mouse samples harvested just before surgery, ESP3: exercise mouse samples harvested on post-surgery day 3, ESP7: exercise mouse samples harvested on post-surgery day 7.

Fig. S3. *Antibiotics reduced gut microbiota.* Nine-week old male mice were treated with antibiotics to eliminate their native gut microbiota. A: bacterial DNA concentrations in feces. Presentation of α diversity is in panels B to D. Presentation of β diversity is in panels E and F. Bacterial abundance is presented in panel G. n = 8 for all panels. C; control, Anti: antibiotics.

Fig. S4. *Transplantation with feces from exercise mice altered gut microbiota.* Nine-week old mice were treated with antibiotics to eliminate their native gut microbiota and then transplanted with feces from exercise mice (ET) or control mice (CT). Presentation of α diversity is in panels A and B. Presentation of β diversity is in panel C. Bacterial abundance is presented in panel D. n = 8 for all panels.

Fig. S5. *Successful establishment of transplanted gut microbiota.* Nine-week old male mice received transplantation of feces from control mice or mice with surgery. Presentation of α diversity is in panels A, B and C. Presentation of β diversity is in panels D and E. Bacterial abundance is presented in panel F. n = 10. CT: control mouse received transplantation of feces from control mice, ST: control mouse received transplantation of feces from surgery mice.

Fig. S6. *Transplantation of gut microbiota from surgery mice induced learning and memory dysfunction.* Nine-week old mice received antibiotic treatment for 7 days (Antibiotic) or transplantation of feces from control mice (Trans-Control) (panels A to C). In another

experiment, nine-week old male mice received transplantation of feces from control mice (Trans-Control) or mice with surgery (Trans-Surgery) (panels D to F). A and D: training sessions of Barnes maze test. B and E: memory assessment of Barnes maze test. C and F: novel object recognition test. Results in panels A and D are mean ± S.D. and results in panels B, C, E and F are median ± interquartile range with presentation of value of individual mouse (n = 11 – 12 for panels A to C, = 16 – 17 for panels D to F).

Fig. S7. *Changes of short chain fatty acids (SCFAs) under various experimental conditions.* Blood and feces were harvested from mice 2 weeks after fecal transplantation (for panels A and D), 7 days after surgery (panel B) or 4 weeks after the onset of exercise protocol (panel C). Results are mean ± S.D. (panels A, C, D) with presentation of value of individual mouse or median ± interquartile range (panel B) with presentation of value of individual mouse (n = 7 for all panels). Trans-Control: mice transplanted with feces from control mice, Trans-Exe: mice transplanted with feces from exercise mice, Exe: exercise, Sur: surgery, NS: normal saline, Val: valeric acid.

Fig. S8. *Intracerebroventricular injection of small dose of valeric acid failed to induce learning and memory dysfunction*. Thirteen-week old male mice received intracerebroventricular injection of normal saline (NS) or valeric acid (Val). A: training sessions of Barnes maze test. B: memory assessment of Barnes maze test. C: novel object recognition test. Results in panel A are mean ± S.D. and results in panels B and C are median ± interquartile range with presentation of value of individual mouse (n = 14 – 15).

Fig. S9. *Exercise via regulating gut microbiota and blood valeric acid concentrations attenuated surgery-induced immune and inflammatory responses.* Hippocampus of 9-week old mice with or without 4-week exercise was harvested at various times after surgery for immunostaining or ELISA. A: representative Iba-1 and C3ar immunostaining images of hippocampus harvested 48 h after surgery. B and C: quantification of Iba-1 and C3ar immunostaining of hippocampus harvested 48 h after surgery. D and E: quantification of C3. F and G: IL-1β quantified by ELISA. H and I: IL-6 quantified by ELISA. Results are mean ± S.D. with presentation of value of individual mouse (n = 6 for panels B and C, = 9 for panels D to F and H, = 10 for panels G and I). Trans-Control: mice transplanted with feces from control mice, Trans-Exe: mice transplanted with feces from exercise mice, Exe: exercise, Sur: surgery, NS: normal saline, Val: valeric acid, post: post-surgery.

Fig. S10. *Exercise via regulating gut microbiota and blood valeric acid concentrations attenuated surgery-induced decrease of brain cell genesis and dendritic arborization.* Brain of 9-week old mice with or without 4-week exercise was harvested 19 days after surgery for immunostaining or Golgi staining. A: representative GFAP and BrdU immunostaining images of hippocampus. B and C: quantification of GFAP and BrdU positively stained cells in the hippocampus. D and E: representative Golgi staining images of hippocampus. F and G: quantification of intersections among the dendritic branches and spine density in the hippocampus. Results are mean ± S.D. with presentation of value of individual mouse (n = 6 for panel B, = 8 for other panels). Trans-Control: mice transplanted with feces from control mice, Trans-Exe: mice transplanted with feces from exercise mice, Exe: exercise, Sur: surgery, NS: normal saline, Val: valeric acid.

Fig. S11. *Exercise attenuated surgery-induced reduction of PSD95 and synapsin 1.* Hippocampus was harvested 2 days after surgery for Western blotting. A and B: representative Western blotting images. C and D: quantification of PSD95 and synapsin 1. Result in panel C is median ± interquartile range with presentation of value of individual mouse and result in panel D is mean ± S.D. with presentation of value of individual mouse (n = 8 for all panels). Trans-Control: mice transplanted with feces from control mice, Trans-Exe: mice transplanted with feces from exercise mice, Exe: exercise, Sur: surgery, Syn-1: synapsin 1.

Fig. S12. *Exercise stabilized gut microbiota of mice with surgery in old mice.* Nineteen-month old male mice with or without exercise for 4 weeks were subjected to left carotid artery exposure (surgery) under isoflurane anesthesia. Presentation of α diversity is in panels A, B, D, E, G, H, J and K. Presentation of β diversity is in panels C, F, I and L. Bacterial abundance is presented in panels M and N. n = 16 for panels A, B, C and M, and = 8 for other panels. OC-OS: old control mouse samples harvested before surgery, OE-OES: old exercise mouse samples harvested before surgery, OS: old surgery mouse samples harvested just before surgery, OSP: old surgery mouse samples harvested on post-surgery day 7, OES: old exercise mouse samples harvested before surgery, OESP: old exercise mouse samples harvested on post-surgery day 7.

Fig. S13. *Exercise attenuated surgery-induced reduction of PSD95 and synapsin 1 in old mice.* Hippocampus was harvested 2 days after surgery for Western blotting. A: representative Western blotting images. B: quantification of PSD95 and synapsin 1. Results are mean ± S.D.

with presentation of value of individual mouse (n = 8). Exe: exercise, Sur: surgery, Syn-1: synapsin 1.

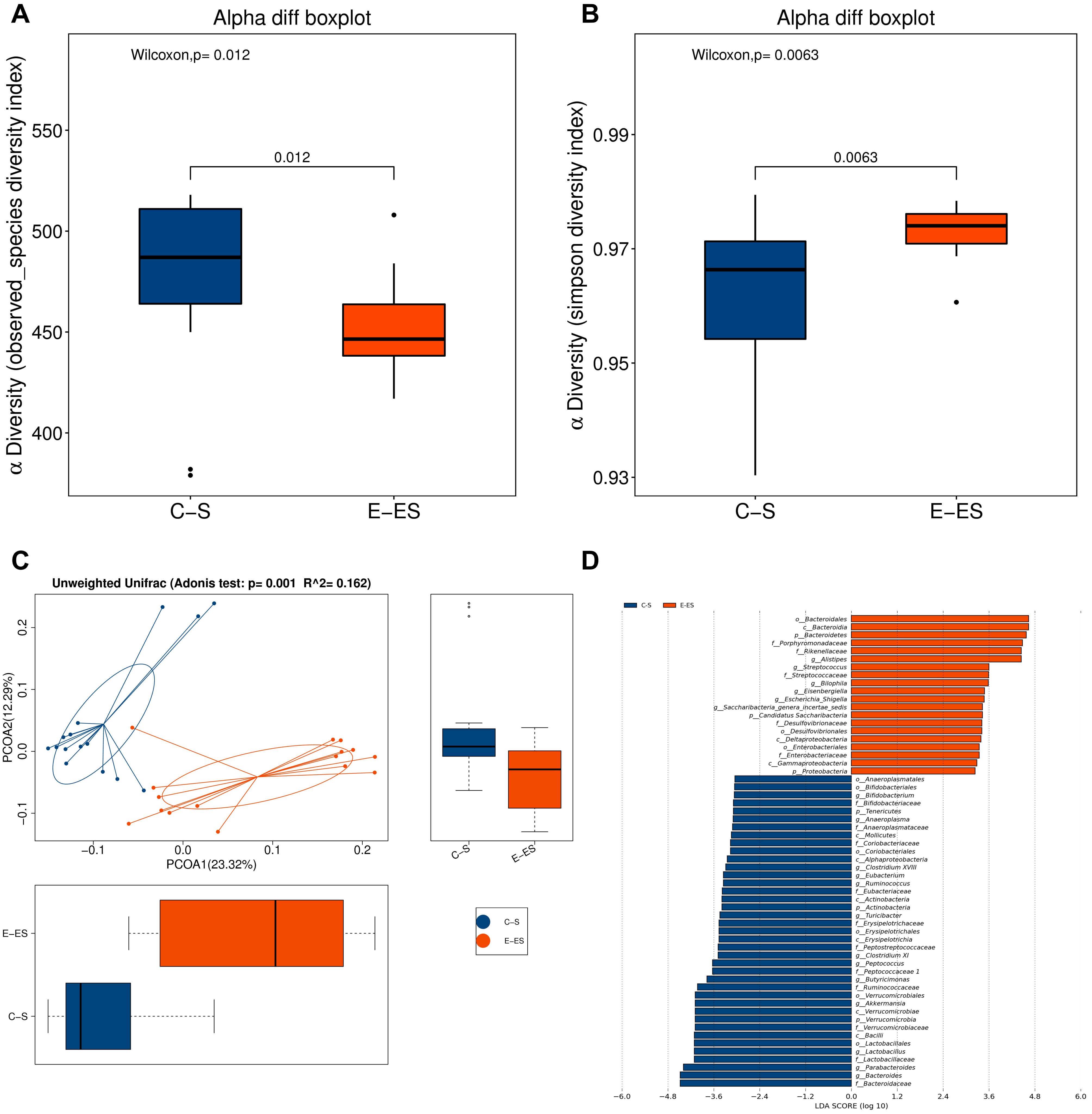

A
Alpha diff boxplot
Wilcoxon,p= 0.012
0.012
α Diversity (observed_species diversity index)
550
500
450
400
C–S
E–ES
B
Alpha diff boxplot
Wilcoxon,p= 0.0063
0.0063
α Diversity (simpson diversity index)
0.99
0.97
0.95
0.93
C–S
E–ES
C
Unweighted Unifrac (Adonis test: p= 0.001 R^2= 0.162)
PCOA2(12.29%)
PCOA1(23.32%)
−0.1
0.0
0.1
0.2
C–S
E–ES
D
C-S
E-ES
o__Bacteroidales
c__Bacteroidia
p__Bacteroidetes
f__Porphyromonadaceae
f__Rikenellaceae
g__Alistipes
g__Streptococcus
f__Streptococcaceae
g__Bilophila
g__Eisenbergiella
g__Escherichia_Shigella
g__Saccharibacteria_genera_incertae_sedis
p__Candidatus Saccharibacteria
f__Desulfovibrionaceae
o__Desulfovibrionales
c__Deltaproteobacteria
o__Enterobacteriales
f__Enterobacteriaceae
c__Gammaproteobacteria
p__Proteobacteria
o__Anaeroplasmatales
o__Bifidobacteriales
g__Bifidobacterium
f__Bifidobacteriaceae
p__Tenericutes
g__Anaeroplasma
f__Anaeroplasmataceae
c__Mollicutes
f__Coriobacteriaceae
o__Coriobacteriales
c__Alphaproteobacteria
g__Clostridium XVIII
g__Eubacterium
g__Ruminococcus
f__Eubacteriaceae
c__Actinobacteria
p__Actinobacteria
g__Turicibacter
f__Erysipelotrichaceae
o__Erysipelotrichales
c__Erysipelotrichia
f__Peptostreptococcaceae
g__Clostridium XI
g__Peptococcus
f__Peptococcaceae 1
g__Butyricimonas
f__Ruminococcaceae
o__Verrucomicrobiales
g__Akkermansia
c__Verrucomicrobiae
p__Verrucomicrobia
f__Verrucomicrobiaceae
c__Bacilli
o__Lactobacillales
g__Lactobacillus
f__Lactobacillaceae
g__Parabacteroides
g__Bacteroides
f__Bacteroidaceae
−6.0 −4.8 −3.6 −2.4 −1.2 0.0 1.2 2.4 3.6 4.8 6.0
LDA SCORE (log 10)

Fig. S1

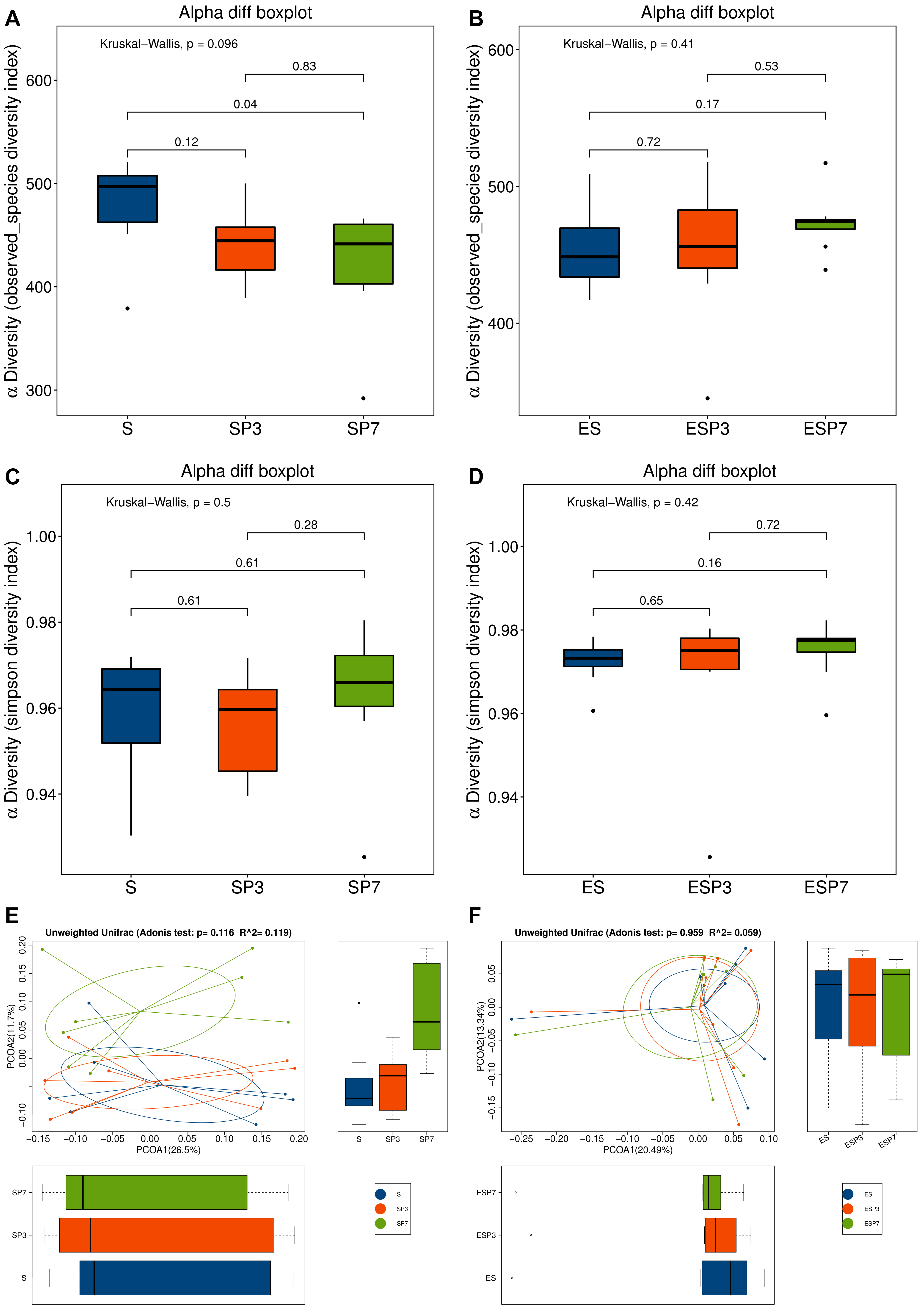
A
Alpha diff boxplot
Kruskal−Wallis, p = 0.096
0.83
0.04
0.12
α Diversity (observed_species diversity index)
600
500
400
300
S
SP3
SP7
B
Alpha diff boxplot
Kruskal−Wallis, p = 0.41
0.53
0.17
0.72
α Diversity (observed_species diversity index)
600
500
400
ES
ESP3
ESP7
C
Alpha diff boxplot
Kruskal−Wallis, p = 0.5
0.28
0.61
0.61
α Diversity (simpson diversity index)
1.00
0.98
0.96
0.94
S
SP3
SP7
D
Alpha diff boxplot
Kruskal−Wallis, p = 0.42
0.72
0.16
0.65
α Diversity (simpson diversity index)
1.00
0.98
0.96
0.94
ES
ESP3
ESP7
E
Unweighted Unifrac (Adonis test: p= 0.116 R^2= 0.119)
PCOA1(26.5%)
PCOA2(11.7%)
S
SP3
SP7
F
Unweighted Unifrac (Adonis test: p= 0.959 R^2= 0.059)
PCOA1(20.49%)
PCOA2(13.34%)
ES
ESP3
ESP7

Fig. S2

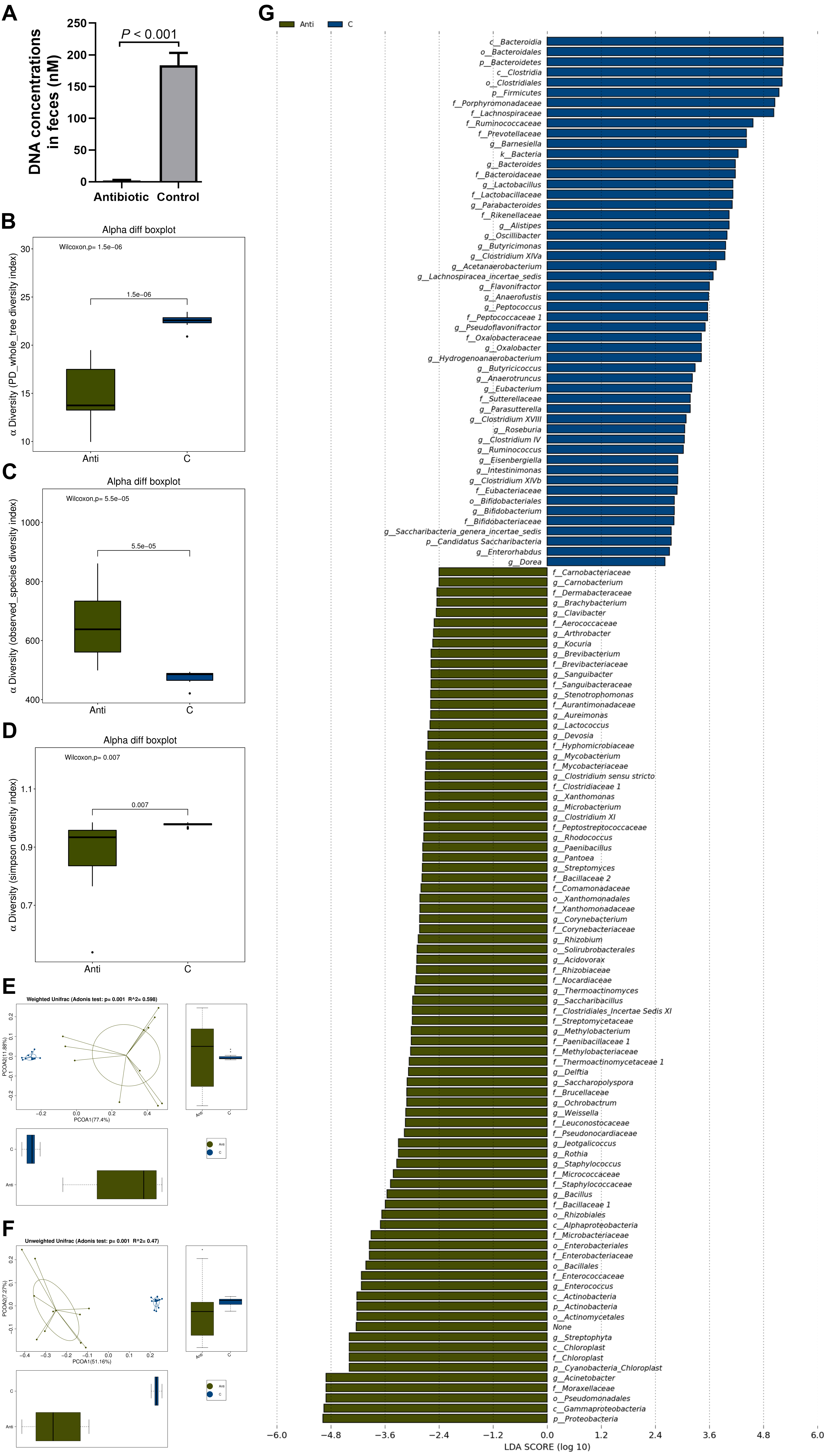
A
DNA concentrations in feces (nM)
P < 0.001
250
200
150
100
50
0
Antibiotic
Control
B
Alpha diff boxplot
Wilcoxon,p= 1.5e−06
1.5e−06
α Diversity (PD_whole_tree diversity index)
30
25
20
15
10
Anti
C
C
Alpha diff boxplot
Wilcoxon,p= 5.5e−05
5.5e−05
α Diversity (observed_species diversity index)
1000
800
600
400
Anti
C
D
Alpha diff boxplot
Wilcoxon,p= 0.007
0.007
α Diversity (simpson diversity index)
1.1
0.9
0.7
Anti
C
E
Weighted Unifrac (Adonis test: p= 0.001 R^2= 0.598)
PCOA1(77.4%)
PCOA2(11.88%)
F
Unweighted Unifrac (Adonis test: p= 0.001 R^2= 0.47)
PCOA1(51.16%)
PCOA2(7.27%)
G
Anti
C
c__Bacteroidia
o__Bacteroidales
p__Bacteroidetes
c__Clostridia
o__Clostridiales
p__Firmicutes
f__Porphyromonadaceae
f__Lachnospiraceae
f__Ruminococcaceae
f__Prevotellaceae
g__Barnesiella
k__Bacteria
g__Bacteroides
f__Bacteroidaceae
g__Lactobacillus
f__Lactobacillaceae
g__Parabacteroides
f__Rikenellaceae
g__Alistipes
g__Oscillibacter
g__Butyricimonas
g__Clostridium XlVa
g__Acetanaerobacterium
g__Lachnospiracea_incertae_sedis
g__Flavonifractor
g__Anaerofustis
g__Peptococcus
f__Peptococcaceae 1
g__Pseudoflavonifractor
f__Oxalobacteraceae
g__Oxalobacter
g__Hydrogenoanaerobacterium
g__Butyricicoccus
g__Anaerotruncus
g__Eubacterium
f__Sutterellaceae
g__Parasutterella
g__Clostridium XVIII
g__Roseburia
g__Clostridium IV
g__Ruminococcus
g__Eisenbergiella
g__Intestinimonas
g__Clostridium XlVb
f__Eubacteriaceae
o__Bifidobacteriales
g__Bifidobacterium
f__Bifidobacteriaceae
g__Saccharibacteria_genera_incertae_sedis
p__Candidatus Saccharibacteria
g__Enterorhabdus
g__Dorea
f__Carnobacteriaceae
g__Carnobacterium
f__Dermabacteraceae
g__Brachybacterium
g__Clavibacter
f__Aerococcaceae
g__Arthrobacter
g__Kocuria
g__Brevibacterium
f__Brevibacteriaceae
g__Sanguibacter
f__Sanguibacteraceae
g__Stenotrophomonas
f__Aurantimonadaceae
g__Aureimonas
g__Lactococcus
g__Devosia
f__Hyphomicrobiaceae
g__Mycobacterium
f__Mycobacteriaceae
g__Clostridium sensu stricto
f__Clostridiaceae 1
g__Xanthomonas
g__Microbacterium
g__Clostridium XI
f__Peptostreptococcaceae
g__Rhodococcus
g__Paenibacillus
g__Pantoea
g__Streptomyces
f__Bacillaceae 2
f__Comamonadaceae
o__Xanthomonadales
f__Xanthomonadaceae
g__Corynebacterium
f__Corynebacteriaceae
g__Rhizobium
o__Solirubrobacterales
g__Acidovorax
f__Rhizobiaceae
f__Nocardiaceae
g__Thermoactinomyces
g__Saccharibacillus
f__Clostridiales_Incertae Sedis XI
f__Streptomycetaceae
g__Methylobacterium
f__Paenibacillaceae 1
f__Methylobacteriaceae
f__Thermoactinomycetaceae 1
g__Delftia
g__Saccharopolyspora
f__Brucellaceae
g__Ochrobactrum
g__Weissella
f__Leuconostocaceae
f__Pseudonocardiaceae
g__Jeotgalicoccus
g__Rothia
g__Staphylococcus
f__Micrococcaceae
f__Staphylococcaceae
g__Bacillus
f__Bacillaceae 1
o__Rhizobiales
c__Alphaproteobacteria
f__Microbacteriaceae
o__Enterobacteriales
f__Enterobacteriaceae
o__Bacillales
f__Enterococcaceae
g__Enterococcus
c__Actinobacteria
p__Actinobacteria
o__Actinomycetales
None
g__Streptophyta
c__Chloroplast
f__Chloroplast
p__Cyanobacteria_Chloroplast
g__Acinetobacter
f__Moraxellaceae
o__Pseudomonadales
c__Gammaproteobacteria
p__Proteobacteria
−6.0
−4.8
−3.6
−2.4
−1.2
0.0
1.2
2.4
3.6
4.8
6.0
LDA SCORE (log 10)

Fig. S3

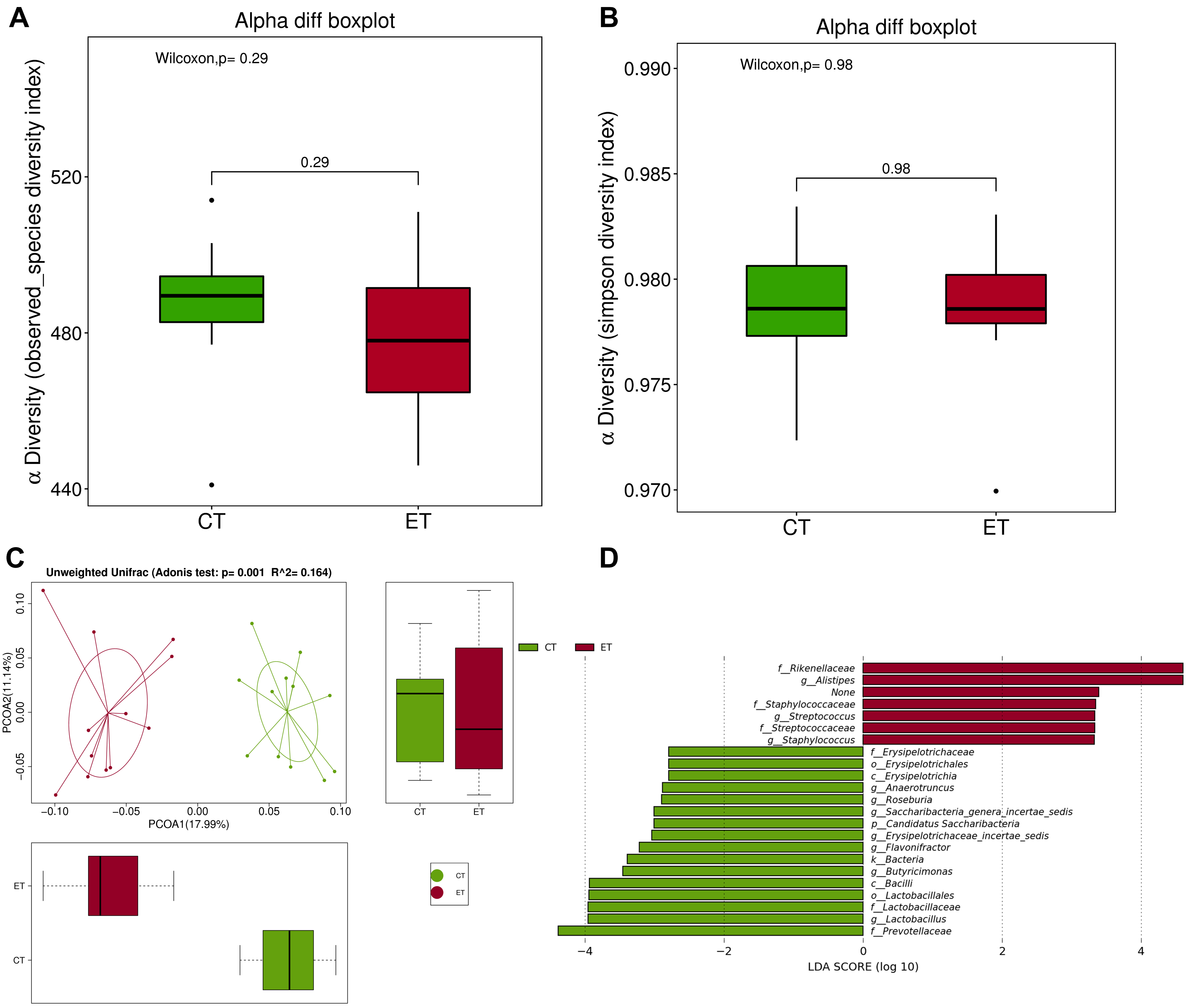
A
Alpha diff boxplot
Wilcoxon,p= 0.29
0.29
α Diversity (observed_species diversity index)
520
480
440
CT
ET
B
Alpha diff boxplot
Wilcoxon,p= 0.98
0.98
α Diversity (simpson diversity index)
0.990
0.985
0.980
0.975
0.970
CT
ET
C
Unweighted Unifrac (Adonis test: p= 0.001 R^2= 0.164)
PCOA2(11.14%)
PCOA1(17.99%)
−0.10
−0.05
0.00
0.05
0.10
CT
ET
D
f__Rikenellaceae
g__Alistipes
None
f__Staphylococcaceae
g__Streptococcus
f__Streptococcaceae
g__Staphylococcus
f__Erysipelotrichaceae
o__Erysipelotrichales
c__Erysipelotrichia
g__Anaerotruncus
g__Roseburia
g__Saccharibacteria_genera_incertae_sedis
p__Candidatus Saccharibacteria
g__Erysipelotrichaceae_incertae_sedis
g__Flavonifractor
k__Bacteria
g__Butyricimonas
c__Bacilli
o__Lactobacillales
f__Lactobacillaceae
g__Lactobacillus
f__Prevotellaceae
−4
−2
0
2
4
LDA SCORE (log 10)

Fig. S4

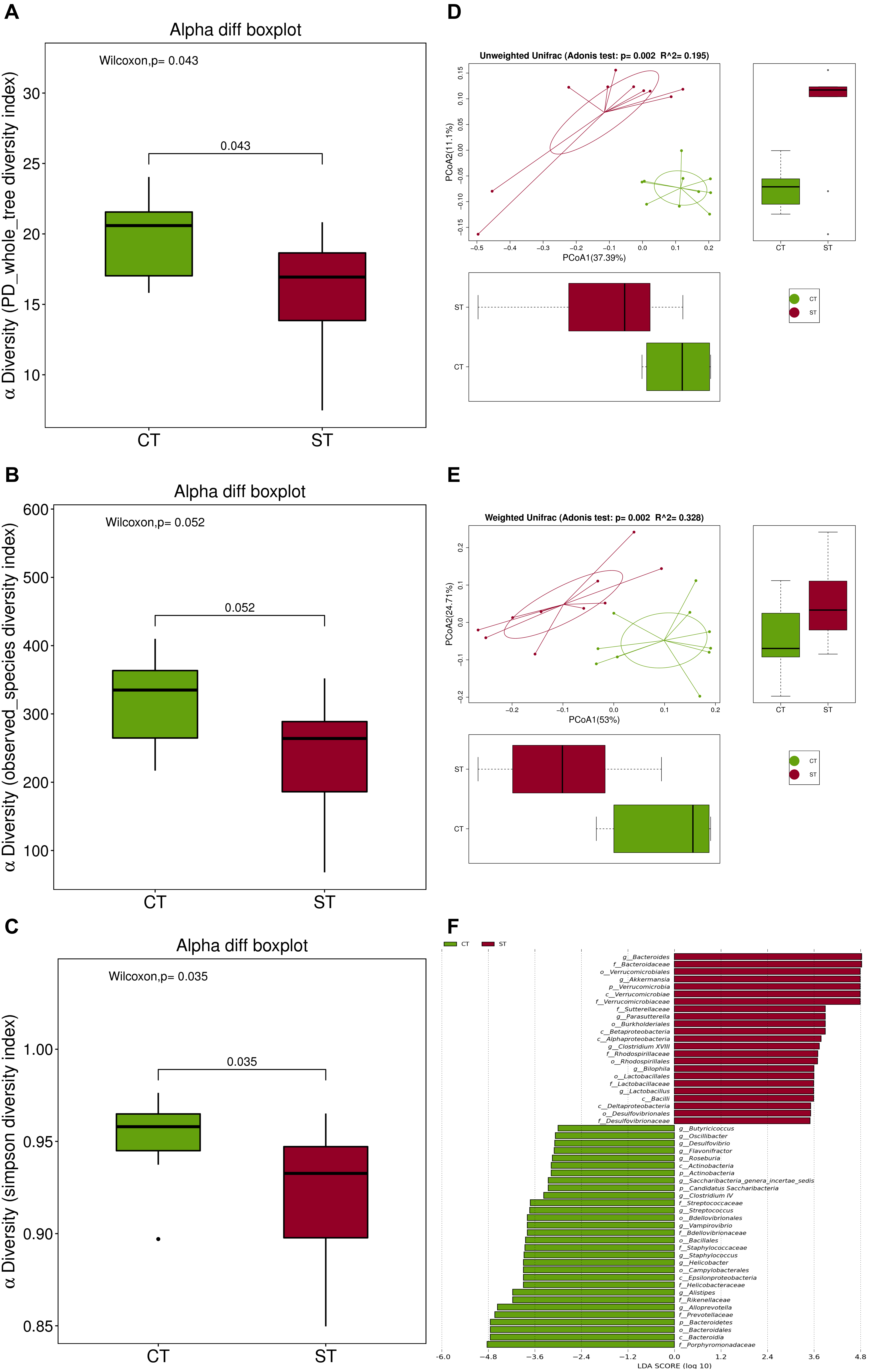
A
Alpha diff boxplot
Wilcoxon,p= 0.043
0.043
α Diversity (PD_whole_tree diversity index)
CT
ST
B
Alpha diff boxplot
Wilcoxon,p= 0.052
0.052
α Diversity (observed_species diversity index)
CT
ST
C
Alpha diff boxplot
Wilcoxon,p= 0.035
0.035
α Diversity (simpson diversity index)
CT
ST
D
Unweighted Unifrac (Adonis test: p= 0.002 R^2= 0.195)
PCoA2(11.1%)
PCoA1(37.39%)
CT
ST
E
Weighted Unifrac (Adonis test: p= 0.002 R^2= 0.328)
PCoA2(24.71%)
PCoA1(53%)
CT
ST
F
CT
ST
g__Bacteroides
f__Bacteroidaceae
o__Verrucomicrobiales
g__Akkermansia
p__Verrucomicrobia
c__Verrucomicrobiae
f__Verrucomicrobiaceae
f__Sutterellaceae
g__Parasutterella
o__Burkholderiales
c__Betaproteobacteria
c__Alphaproteobacteria
g__Clostridium XVIII
f__Rhodospirillaceae
o__Rhodospirillales
g__Bilophila
o__Lactobacillales
f__Lactobacillaceae
g__Lactobacillus
c__Bacilli
c__Deltaproteobacteria
o__Desulfovibrionales
f__Desulfovibrionaceae
g__Butyricicoccus
g__Oscillibacter
g__Desulfovibrio
g__Flavonifractor
g__Roseburia
c__Actinobacteria
p__Actinobacteria
g__Saccharibacteria_genera_incertae_sedis
p__Candidatus Saccharibacteria
g__Clostridium IV
f__Streptococcaceae
g__Streptococcus
o__Bdellovibrionales
g__Vampirovibrio
f__Bdellovibrionaceae
o__Bacillales
f__Staphylococcaceae
g__Staphylococcus
g__Helicobacter
o__Campylobacterales
c__Epsilonproteobacteria
f__Helicobacteraceae
g__Alistipes
f__Rikenellaceae
g__Alloprevotella
f__Prevotellaceae
p__Bacteroidetes
o__Bacteroidales
c__Bacteroidia
f__Porphyromonadaceae
LDA SCORE (log 10)

Fig. S5

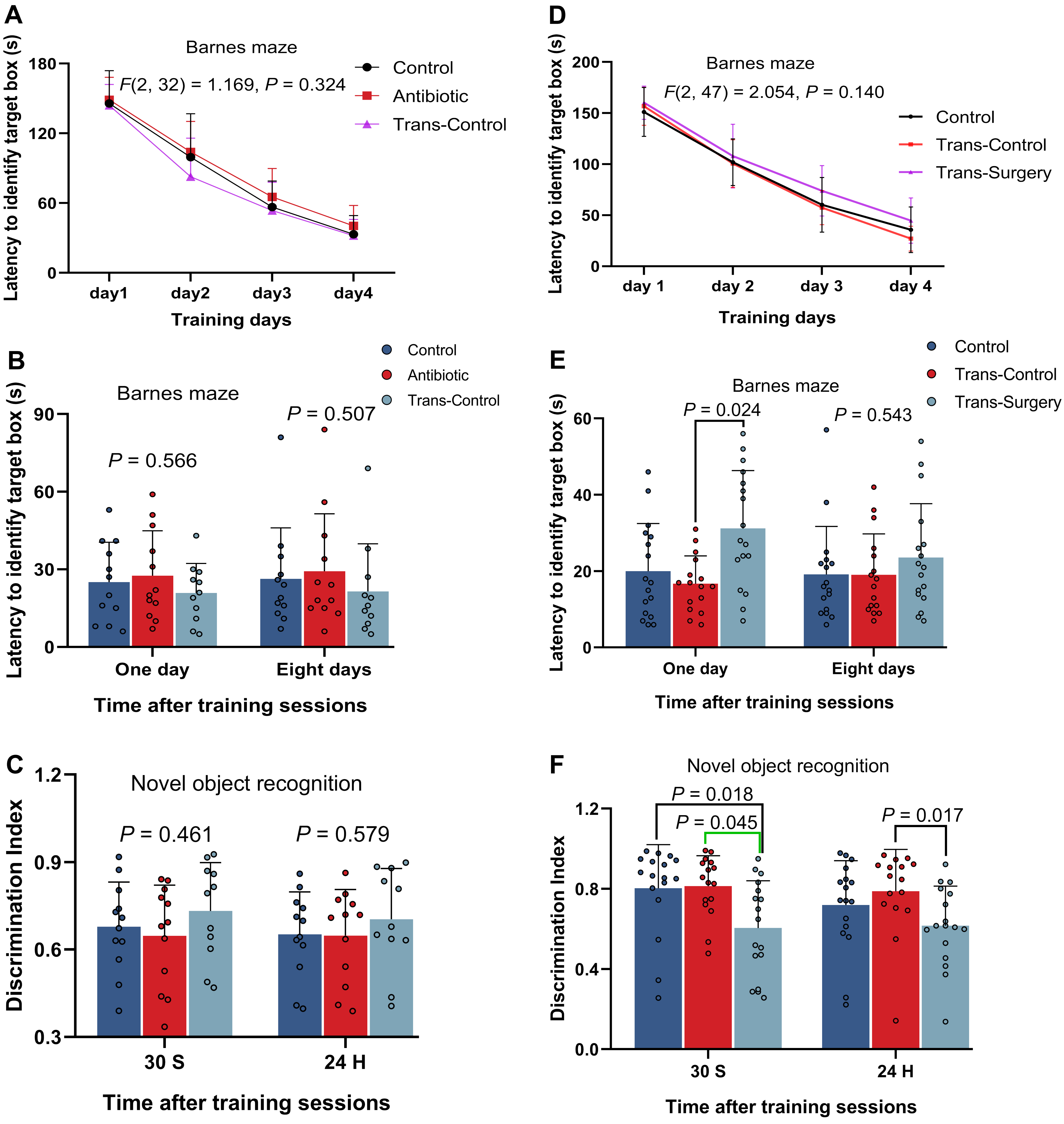
A
Barnes maze
F(2, 32) = 1.169, P = 0.324
Control
Antibiotic
Trans-Control
Latency to identify target box (s)
Training days
B
Barnes maze
P = 0.566
P = 0.507
Control
Antibiotic
Trans-Control
One day
Eight days
Time after training sessions
C
Novel object recognition
P = 0.461
P = 0.579
Discrimination Index
30 S
24 H
Time after training sessions
D
Barnes maze
F(2, 47) = 2.054, P = 0.140
Control
Trans-Control
Trans-Surgery
Training days
E
Barnes maze
P = 0.024
P = 0.543
Control
Trans-Control
Trans-Surgery
One day
Eight days
Time after training sessions
F
Novel object recognition
P = 0.018
P = 0.045
P = 0.017
Discrimination Index
30 S
24 H
Time after training sessions

Fig. S6

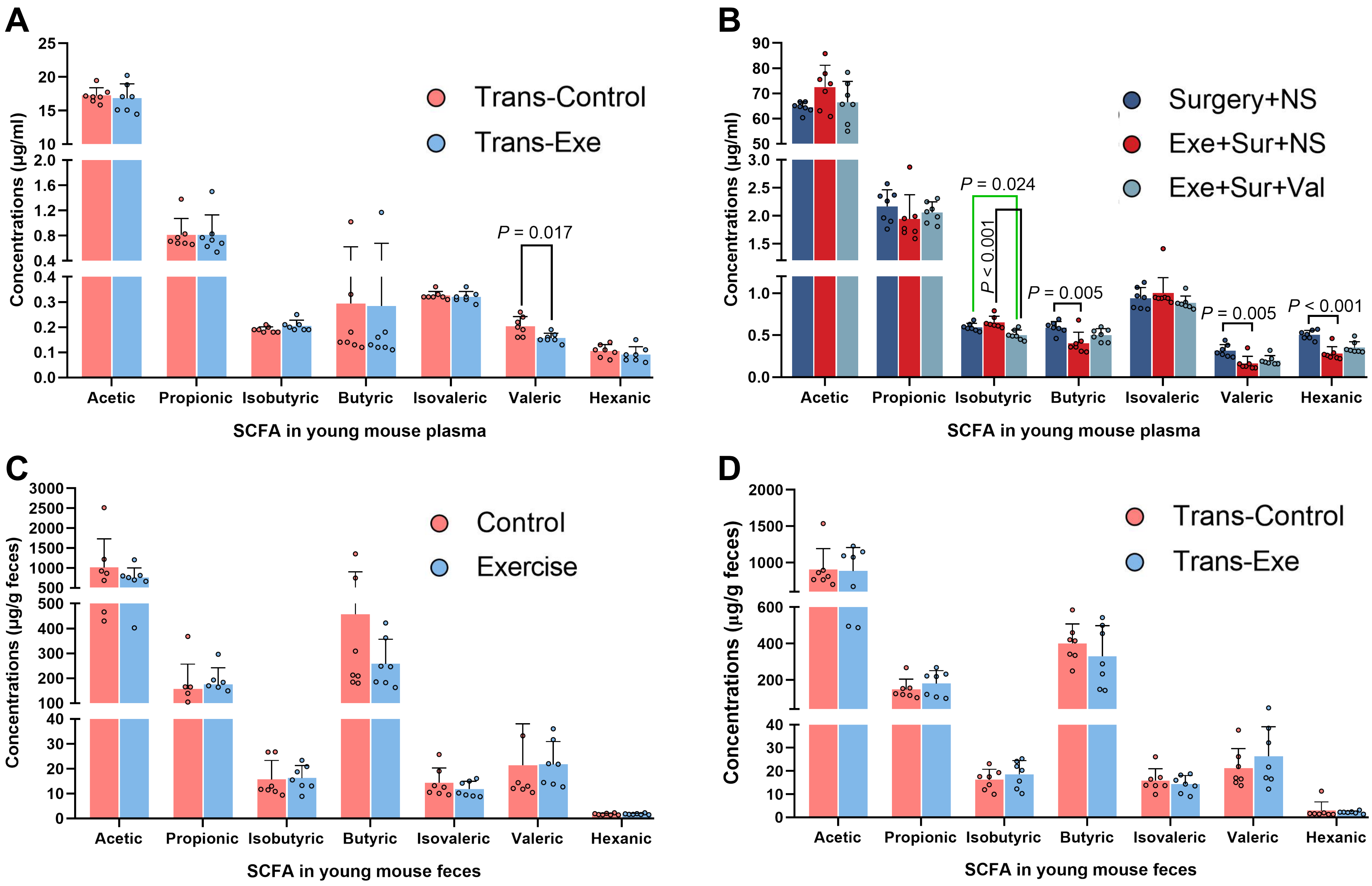
A
Trans-Control
Trans-Exe
Concentrations (μg/ml)
P = 0.017
Acetic
Propionic
Isobutyric
Butyric
Isovaleric
Valeric
Hexanic
SCFA in young mouse plasma
B
Surgery+NS
Exe+Sur+NS
Exe+Sur+Val
P = 0.024
P < 0.001
P = 0.005
P = 0.005
P < 0.001
SCFA in young mouse plasma
C
Control
Exercise
Concentrations (μg/g feces)
SCFA in young mouse feces
D
Trans-Control
Trans-Exe
SCFA in young mouse feces

Fig. S7

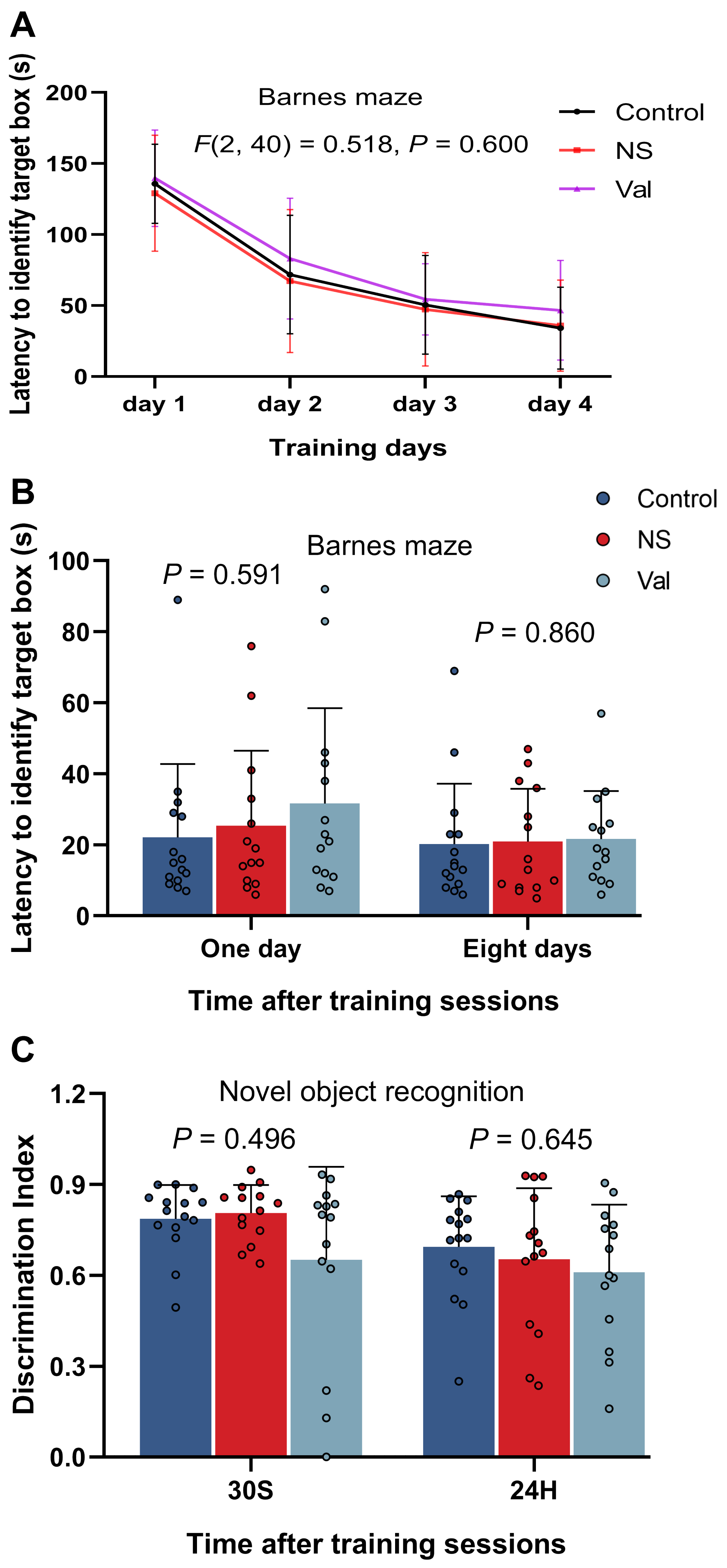
A
Barnes maze
F(2, 40) = 0.518, P = 0.600
Control
NS
Val
Latency to identify target box (s)
200
150
100
50
0
day 1
day 2
day 3
day 4
Training days
B
Control
NS
Val
Barnes maze
P = 0.591
P = 0.860
Latency to identify target box (s)
100
80
60
40
20
0
One day
Eight days
Time after training sessions
C
Novel object recognition
P = 0.496
P = 0.645
Discrimination Index
1.2
0.9
0.6
0.3
0.0
30S
24H
Time after training sessions

Fig. S8

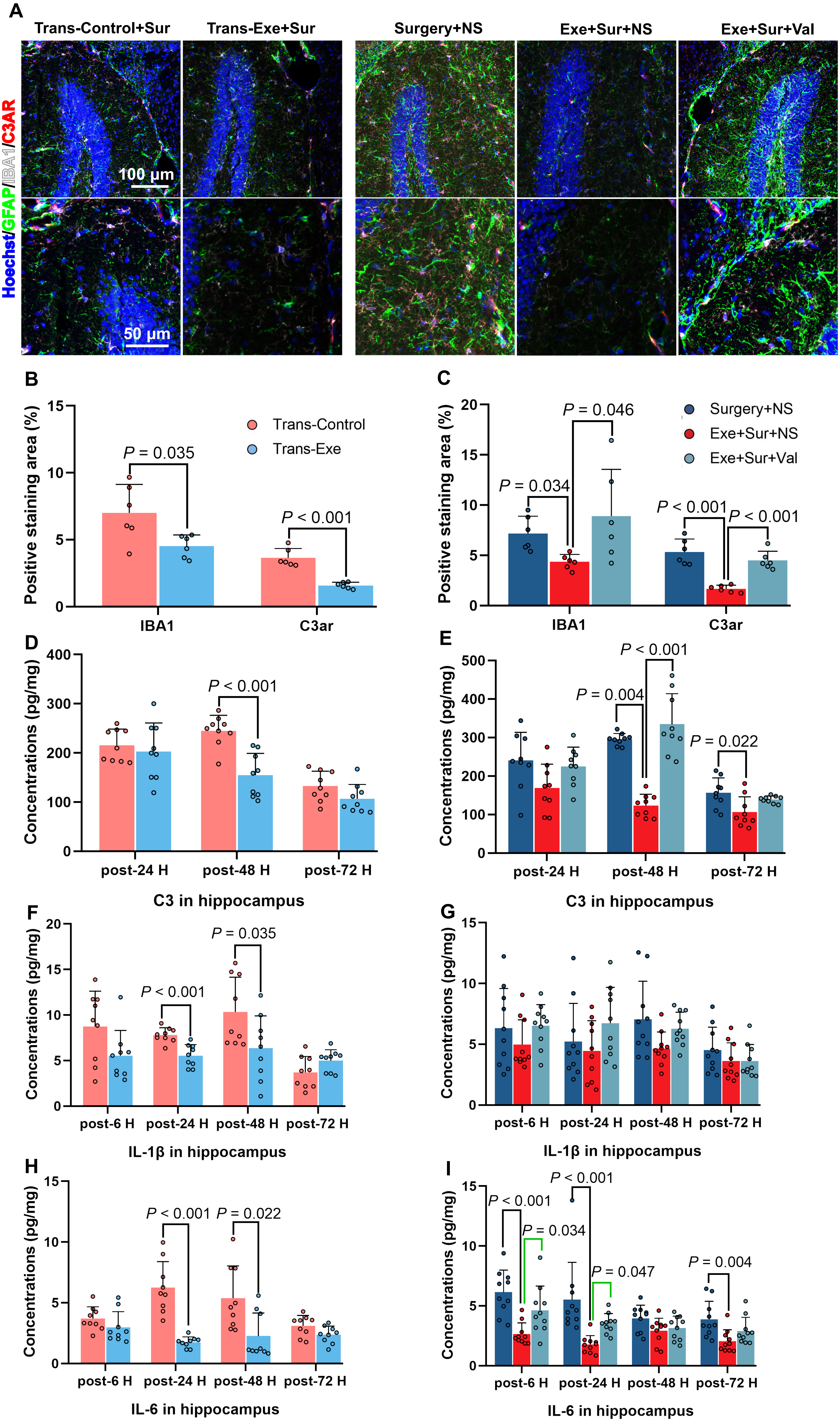

A
Trans-Control+Sur
Trans-Exe+Sur
Surgery+NS
Exe+Sur+NS
Exe+Sur+Val
Hoechst/GFAP/IBA1/C3AR
100 μm
50 μm
B
Positive staining area (%)
Trans-Control
Trans-Exe
P = 0.035
P < 0.001
IBA1
C3ar
C
Surgery+NS
Exe+Sur+NS
Exe+Sur+Val
P = 0.046
P = 0.034
P < 0.001
P < 0.001
D
Concentrations (pg/mg)
P < 0.001
post-24 H
post-48 H
post-72 H
C3 in hippocampus
E
P < 0.001
P = 0.004
P = 0.022
C3 in hippocampus
F
P = 0.035
P < 0.001
post-6 H
IL-1β in hippocampus
G
IL-1β in hippocampus
H
P < 0.001
P = 0.022
IL-6 in hippocampus
I
P < 0.001
P < 0.001
P = 0.034
P = 0.047
P = 0.004
IL-6 in hippocampus

Fig. S9

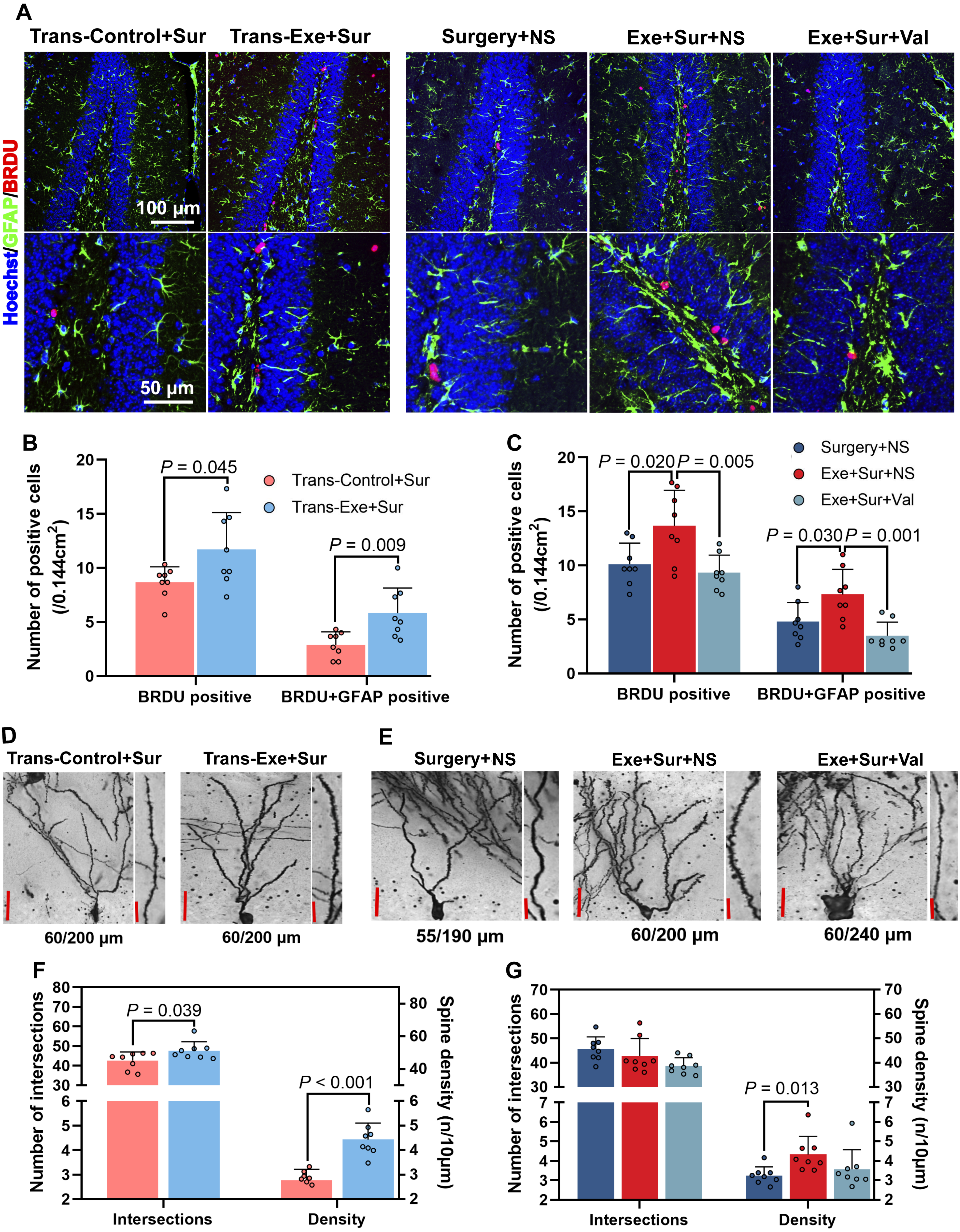

A
Trans-Control+Sur
Trans-Exe+Sur
Surgery+NS
Exe+Sur+NS
Exe+Sur+Val
Hoechst/GFAP/BRDU
100 μm
50 μm
B
Number of positive cells (/0.144cm^2)
P = 0.045
P = 0.009
Trans-Control+Sur
Trans-Exe+Sur
BRDU positive
BRDU+GFAP positive
C
P = 0.020
P = 0.005
P = 0.030
P = 0.001
Surgery+NS
Exe+Sur+NS
Exe+Sur+Val
BRDU positive
BRDU+GFAP positive
D
Trans-Control+Sur
Trans-Exe+Sur
60/200 μm
60/200 μm
E
Surgery+NS
Exe+Sur+NS
Exe+Sur+Val
55/190 μm
60/200 μm
60/240 μm
F
Number of intersections
Spine density (n/10μm)
P = 0.039
P < 0.001
Intersections
Density
G
Number of intersections
Spine density (n/10μm)
P = 0.013
Intersections
Density

Fig. S10

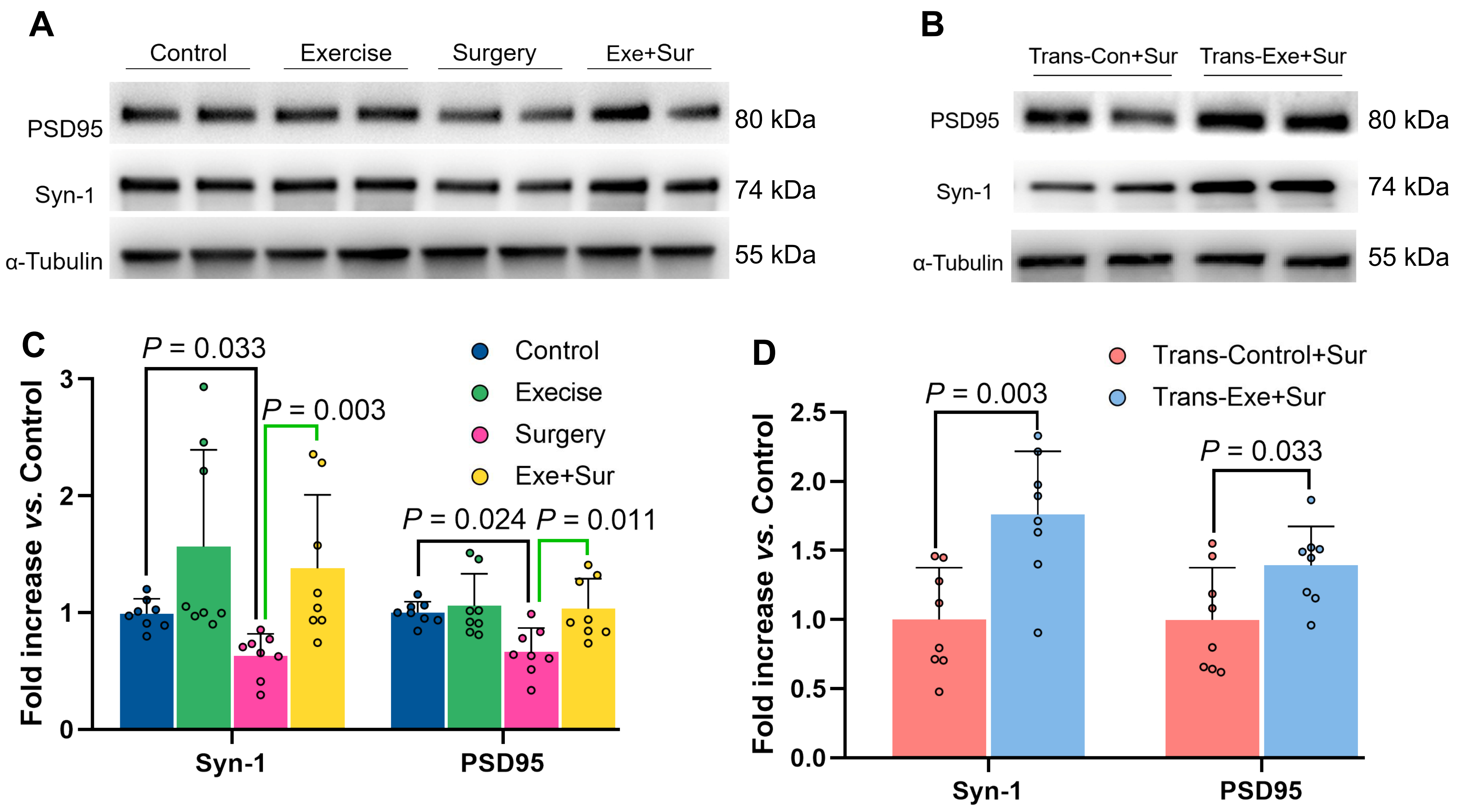

A
Control
Exercise
Surgery
Exe+Sur
PSD95
Syn-1
α-Tubulin
80 kDa
74 kDa
55 kDa
B
Trans-Con+Sur
Trans-Exe+Sur
PSD95
Syn-1
α-Tubulin
80 kDa
74 kDa
55 kDa
C
Fold increase vs. Control
P = 0.033
P = 0.003
P = 0.024
P = 0.011
Control
Execise
Surgery
Exe+Sur
Syn-1
PSD95
D
Fold increase vs. Control
P = 0.003
P = 0.033
Trans-Control+Sur
Trans-Exe+Sur
Syn-1
PSD95

Fig. S11

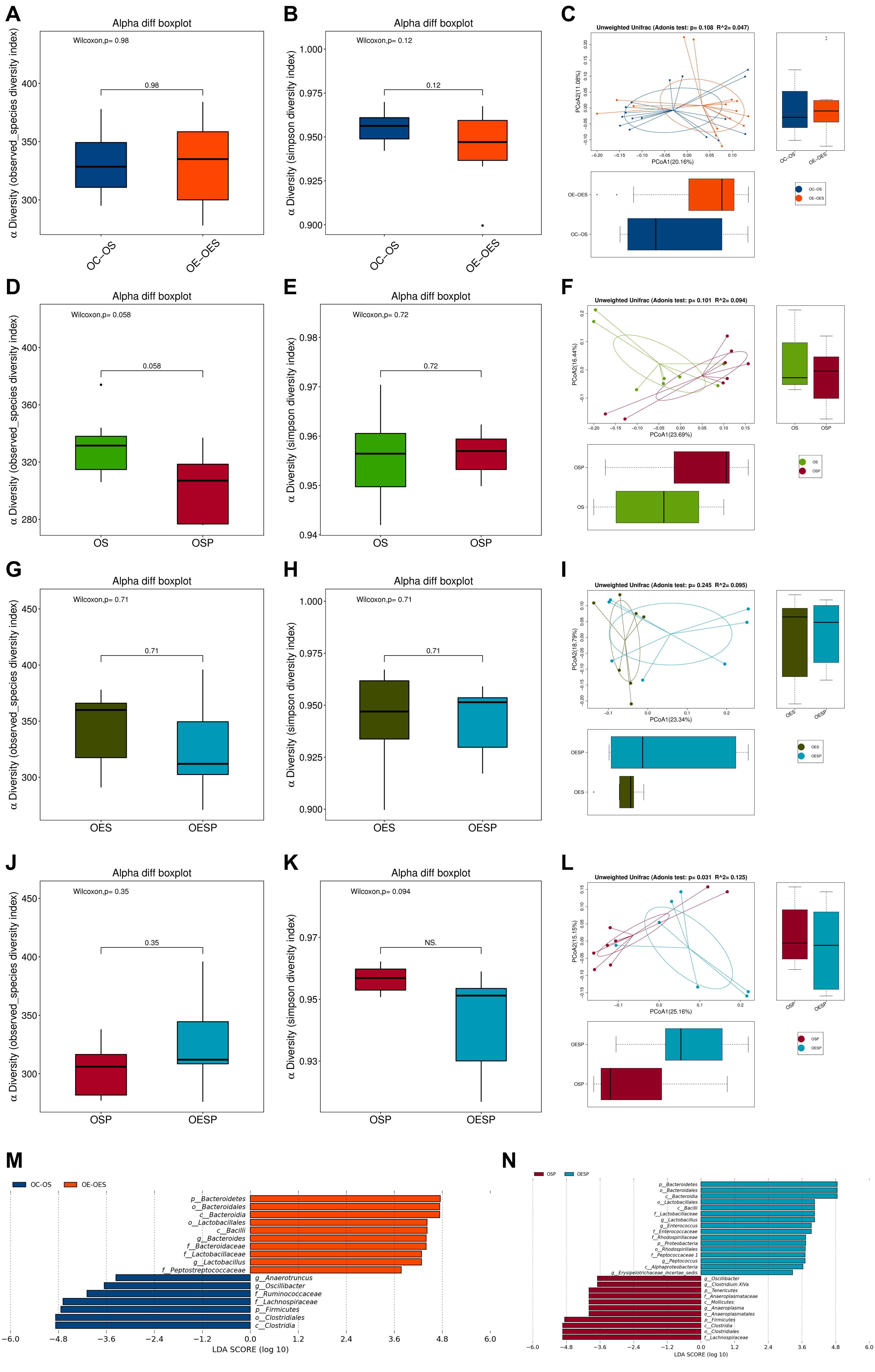
A
Alpha diff boxplot
Wilcoxon,p= 0.98
B
Alpha diff boxplot
Wilcoxon,p= 0.12
C
D
Alpha diff boxplot
Wilcoxon,p= 0.058
E
Alpha diff boxplot
Wilcoxon,p= 0.72
F
G
Alpha diff boxplot
Wilcoxon,p= 0.71
H
Alpha diff boxplot
Wilcoxon,p= 0.71
I
J
Alpha diff boxplot
Wilcoxon,p= 0.35
K
Alpha diff boxplot
Wilcoxon,p= 0.094
L
M
N
LDA SCORE (log 10)

Fig. S12

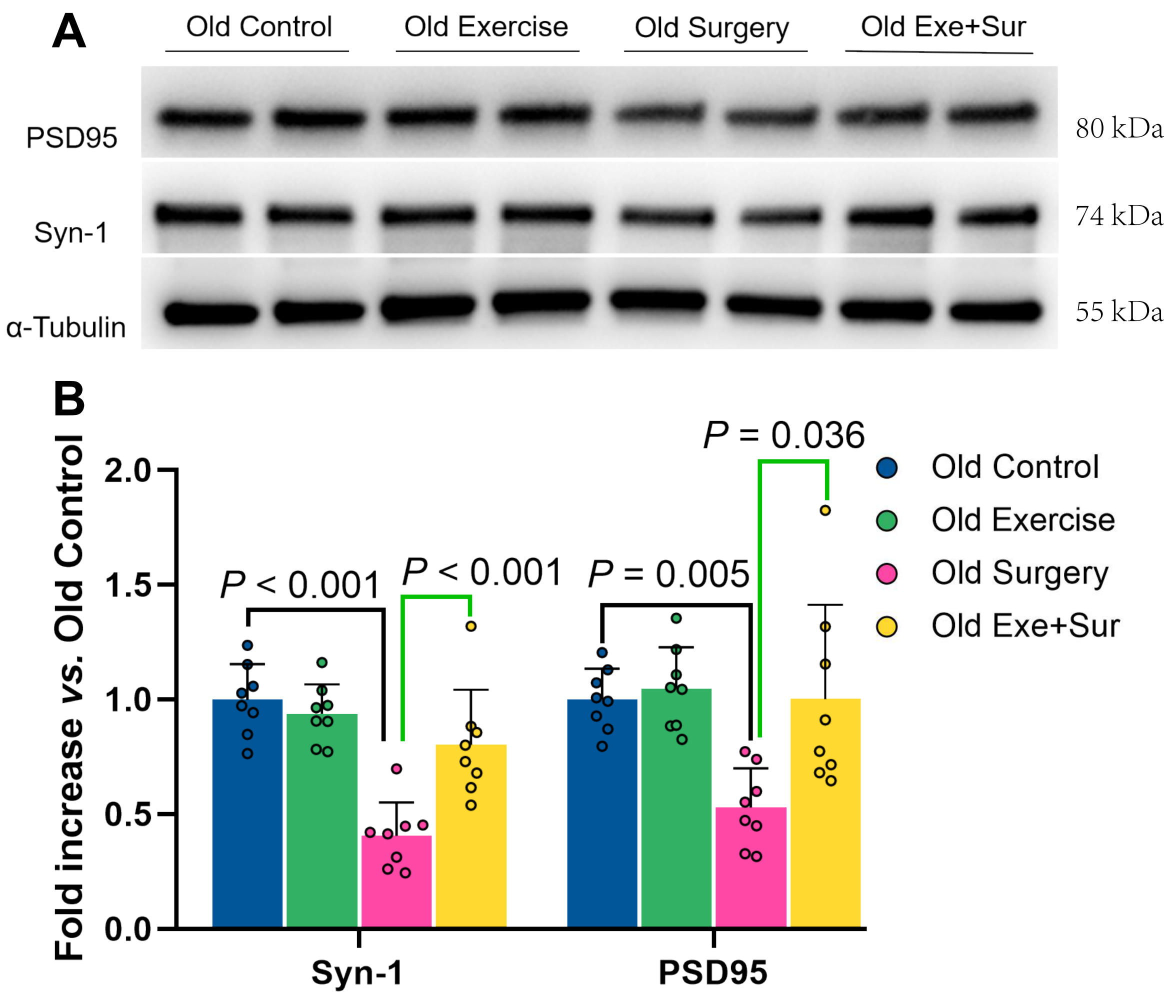
A
Old Control
Old Exercise
Old Surgery
Old Exe+Sur
PSD95
Syn-1
α-Tubulin
80 kDa
74 kDa
55 kDa
B
Fold increase vs. Old Control
2.0
1.5
1.0
0.5
0.0
P < 0.001
P < 0.001
P = 0.005
P = 0.036
Syn-1
PSD95
Old Control
Old Exercise
Old Surgery
Old Exe+Sur

Fig. S13
